# Anomalous dynamic response and micro-zonation of saline sabkha soils of eastern Saudi Arabia using 3D seismic behavior modelling

**DOI:** 10.1038/s41598-025-31302-9

**Published:** 2025-12-11

**Authors:** Muhammad Fayyaz, Naser A. Al-Shayea, Muhammad Abdul Waheed, Sahel N. Abduljauwad, Habib-ur-Rehman Ahmed

**Affiliations:** 1 Riyadh Geotechnique and Foundations (RGF), Dammam, Saudi Arabia; 2 Civil and Environmental Engineering Department, King Fahd University of Petroleum and Minerals (KFUPM), Dhahran, Saudi Arabia

**Keywords:** Seismic response, Sabkha soils, 3-D dynamic modelling and simulation, Micro-zonation, Pore fluid effects, Liquefaction potential, Natural hazards, Civil engineering

## Abstract

The available seismic response parameters do not accurately represent the unusual behavior of sabkha soils; the weak soils with high salt content. This study aimed at developing seismic response parameters for sabkha soils in Ras Al-Khair Industrial City, Saudi Arabia. These sabkhas revealed an anomalous response to geophysical and dynamic tests. The methodology involved analysis of extensive available data to come up with micro-zonation based on subsurface strata. Field and laboratory explorations were conducted, and 3-D seismic response was numerical simulated. This study identified an anomalous dynamic response of sabkha soil, as it has higher shear modulus. The spectral accelerations values, for both short-period and long-period, have been found to differ from the values specified in local codes. Scientific contributions include the localized liquefaction potential along the boundary of the contrasting strata. The site-specific seismic response is found to be not only dependent on the structure and fabric of the soil, but also highly affected by the pore fluid. Originality appears in utilizing 3-D modelling/ simulations to produce liquefaction hazard maps. It is concluded that 3-D modelling/simulations lead to a more realistic representation of ground conditions. The seismic parameters are recommended to be revised based on 3-D modelling that considers the type of pore fluids.

## Introduction

The east coast of Saudi Arabia is characterized by sabkha soils. Sabkha is an Arabic term for flat salt-crusted deposits consisting of wet, loose silty sand and/or soft clay. It is identified by high concentrations of salt, shallow water tables, and low shear strength^1–4^. The eastern coast is the home for large industrial facilities, including Ras Al Khair Industrial City (RIC) which is being built on sabkha deposits. Due to their anomalous behavior under various loading conditions, the development of design parameters for these sabkhas has presented challenges for design engineers. Given the increasing likelihood and intensity of earthquakes in the region^5–7^, determining the dynamic response characteristics of sabkhas has also become critical. The precise assessment of spectral response parameters of sabkhas will lead to more realistic design of structures against earthquakes. Moreover, the potential of liquefaction of these layers under earthquake loading poses a serious threat to the stability of the foundations placed on these sabkhas^8–14^. Therefore, performing a realistic modeling for the seismic behavior of sabkhas is essential for the structural and geotechnical design of facilities in the region.

Local research on the determination of seismic response parameters and behavior can be divided into macro-zonation and micro-zonation categories. The macro-zonation studies in the region^16–20^ are primarily based on a generalized soil profile for the entire area, without considering local variations in geology, type of subsurface strata, topography, and boundaries of various local lithological units. On the other hand, micro-zonation considers these variations. Consideration of local variations make the distinction between micro- and macro-zonation. Some micro-zonation studies^5,7,8,21–26^ considered local subsurface stratum variations, but no recommendations were provided for the seismic response parameters of these micro-zones in most of these studies. Moreover, there is still a gap that makes these micro and macro-zonation studies of limited capabilities to model soil behavior. This gap is due to the use of 1-D seismic analysis, and to ignore local site variations. Recent studies^27–30^ have confirmed the differences between 1-D and 3-D seismic modeling for evaluating seismic parameters. The significant advantage of 3-D modelling over 1-D modelling is the ability to capture two aspects on the seismic response parameters. These are the effects of irregular stratigraphy and topography, as well as the boundaries of contrasting lithologies. Moreover, the 3-D modelling can capture the basin effect and 3-D wave dispersion effects; and thus, closely simulate the real behavior during a seismic activity. These essential mechanisms cannot be captured in the 1-D modelling.

To close this gap, this study was initiated to improve the design and construction of industrial facilities in the area. It aimed at performing 3-D seismic behavior modeling and micro-zonation for different sabkhas in RIC. The methodology includes analysis of geological, lithological, and tectonic data of the study area; performing field and laboratory testing to acquire dynamic design parameters; and modeling the seismic behavior of the subsurface strata profiles using FEM-based software. This research resulted in seismic micro-zonation and liquefaction hazard maps for the study area at RIC.

## Background

### Geology of the area

The geology of the study area consists of Quaternary sabkhas and aeolian sand deposits underlain by the Hadrukh Formation of early Miocene age. The Hadrukh Formation comprises a mixed succession of siliciclastic, carbonates, and evaporite deposits. It rests on the Paleogene, Dammam, Rus, and Umm Er Radhuma formations, and is bounded at the top by the early Middle Miocene Dam Formation. The Upper Hadrukh Member comprises calcareous sandstones and gypsiferous marls with intercalated sandy limestone. The depositional environment is a marginal marine and tidally influenced fluvial setting, associated with coastal sabkhas. The soil deposits in the region are well known for their heterogeneity^31^.

**Fig. 1:**
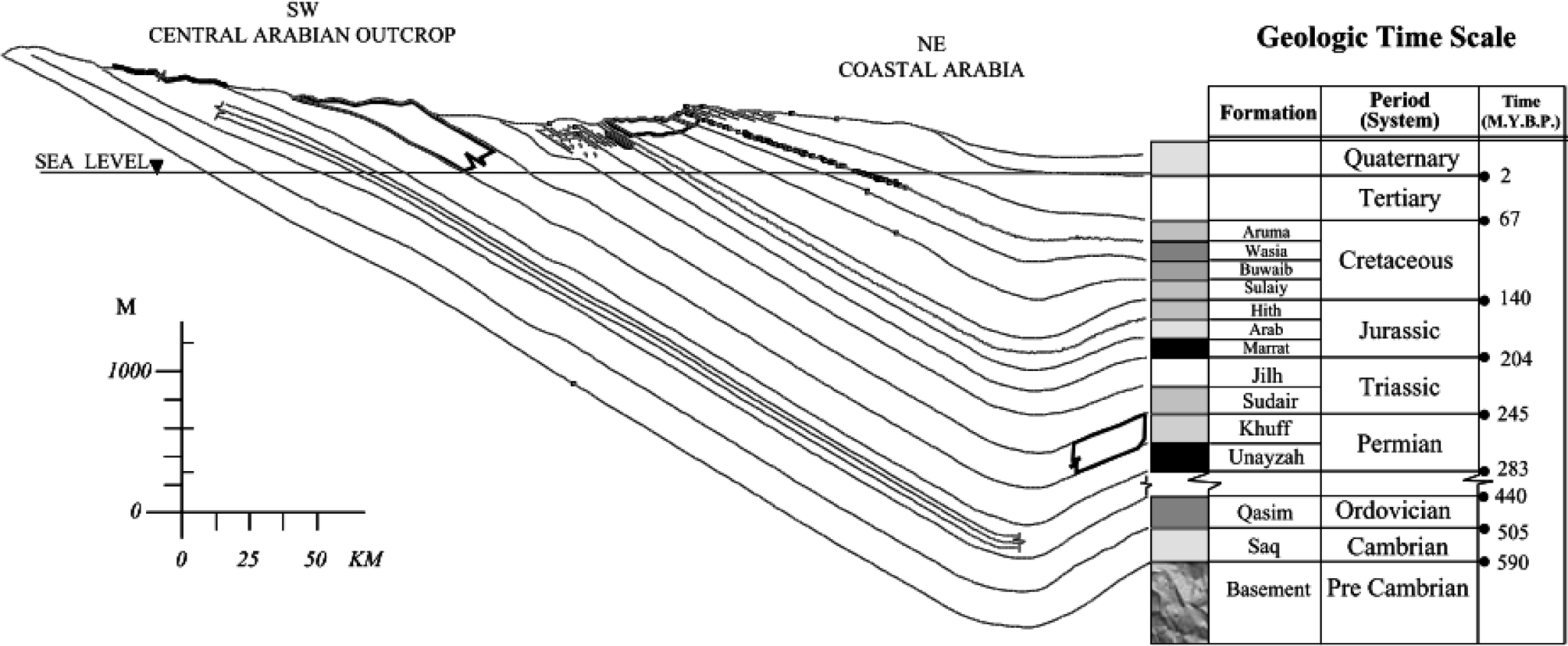
The structural geology of the sedimentary rock formations in the region^32^.

In addition to the Miocene Hadrukh and Quaternary sabkhas, there are wind-blown Quaternary sand deposits. A general trend of the sedimentary rock formations in the region is shown in Fig. 1. The structural geology for these formations indicates that they outcrop at various places in the central Arabia, with an altitude reaching hundreds of meters above the sea level, and they dip toward the east to a depth of about two to four thousand meters below the sea level at the east coast^32^.

### Lithology of the site

For the subsurface strata at the site, data was acquired from several hundred boreholes drilled in the area for various projects. Data on subsurface boreholes, crosshole seismic tests, and multichannel analysis of surface waves (MASW) survey were acquired from the central database of Riyadh Geotechnique and Foundations Co. (RGF). The collected information was analyzed, synthesized and categorized. Based on that, the shallow subsurface soil layers can be essentially grouped into lithological units. The lithological units could generally be categorized into broad groups with several subgroups.

### Zonation of the site

**Fig. 2:**
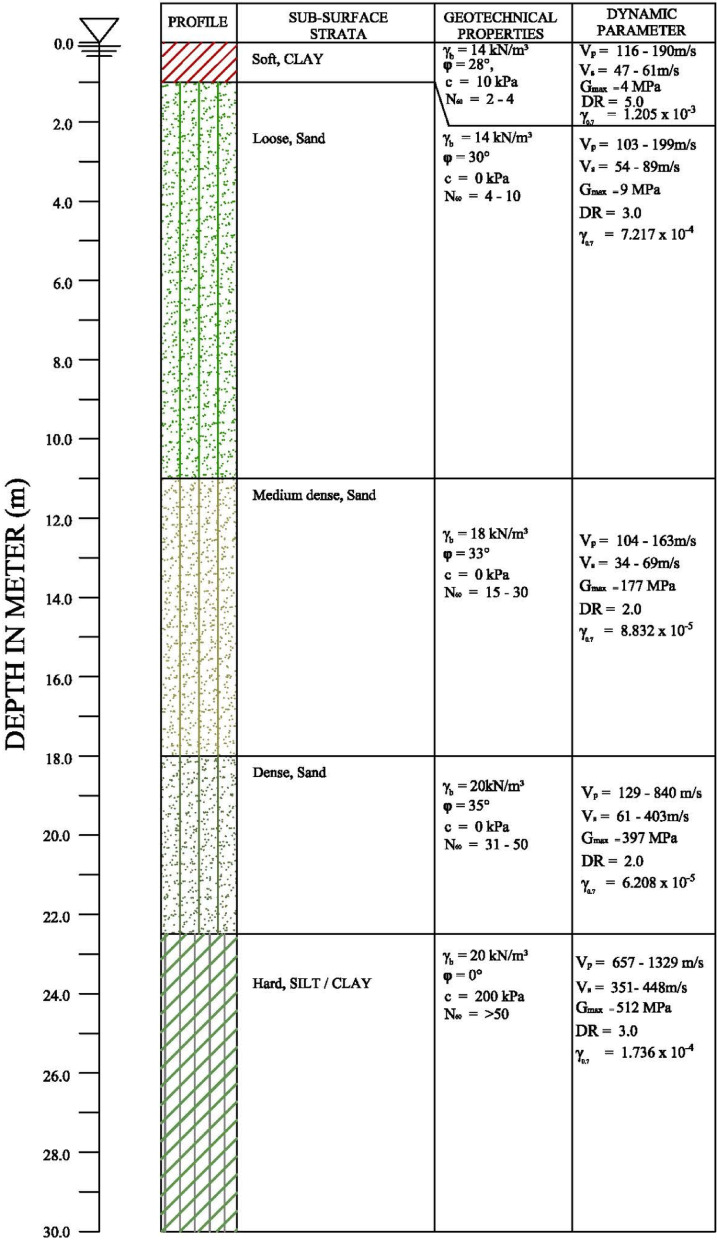
Typical profile of the sandy sabkha zone.

Based on the lithological units, the subsurface strata are divided into two main zones. These are sabkha zone and non-sabkha zone. The sabkha zone is divided into two zones; sandy sabkha and clayey sabkha. Figure 2 shows a typical profile for the sandy sabkha zone, which is selected because of its high practical relevancy. Table 1 provides statistical analysis for the results of the SPT and the seismic tests. Similar profiles and statistical analysis for other zones are available, but not presented herein for size limitations. The subsurface strata variations in each zone are explained as follows.

**Table 1:** Statistics of various field test for sandy sabkha zone.

Layer	Item	SPT (N value)	V_p_ (m/s)	V_s_ (m/s)
Upper	Number of tests	500	13	13
	Range	4–10	116–190	47–61
	Average	6	151	55
	Standard deviation	3	26	19
Middle-upper	Number of tests	450	10	10
	Range	15–30	104–163	34–69
	Average	23	142	61
	Standard deviation	7	23	15
Middle-lower	Number of tests	375	6	6
	Range	31–50	129–840	61–403
	Average	39	743	370
	Standard deviation	11	62	24
Lower	Number of tests	300	10	10
	Range	> 50	657–1329	351–448
	Average	> 50	1054	401
	Standard deviation	> 50	119	27

#### Sabkha zone

This zone is characterized by the salt-rich soils known as “sabkha”. This zone is divided into two sub-groups sandy sabkha and clayey sabkha. The sandy sabkha zone comprise loose sand at the surface having a thickness of 2.0–11.0 m. The soil layers below that consist of medium-dense to very dense sand, extending to a depth of 20–22 m. The clayey sabkha zone consists of soft clays at the surface, ranging from 1.0 to 4.5 m in thickness, underlain by medium-dense to very-dense sand layers extending to a depth of 18–20 m. Hard clay layers of the Hadrukh formation underlie the very dense layers in both zones. These layers are similar and comparable to those reported for another sabkha deposit in the vicinity of the study area, which was mapped using GIS^33^. The combined effects of clay and water contents on the behavior of local soils were studied^34^ and modelled^35^. The influence of calcium sulphate minerals on the swelling of clay was also investigated^36^.

#### Non-sabkha zone

This zone comprises wind-blown aeolian sands of varying density overlying the hard clays of the Hadrukh formation. The sand is a medium-dense from the surface to about 6.0 m depth, followed by very dense sand down to the top of the hard clay strata. The hard clay is termed as the bedrock, because it causes refusal for SPT tests.

### Tectonics and seismology of the region

**Fig. 3:**
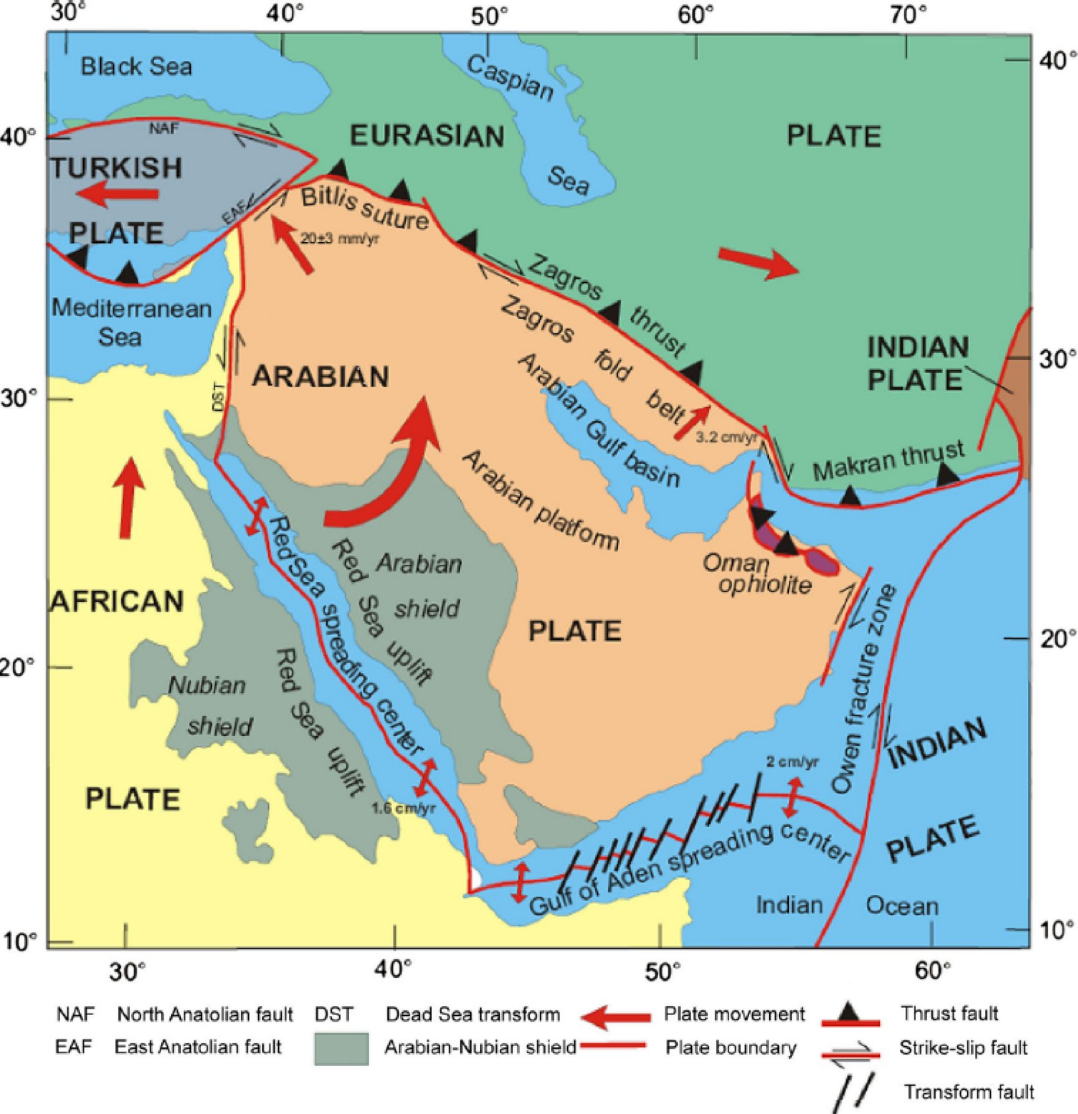
Location of major tectonic elements of the Arabian Plate and Eurasian Plate^6^.

**Fig. 4:**
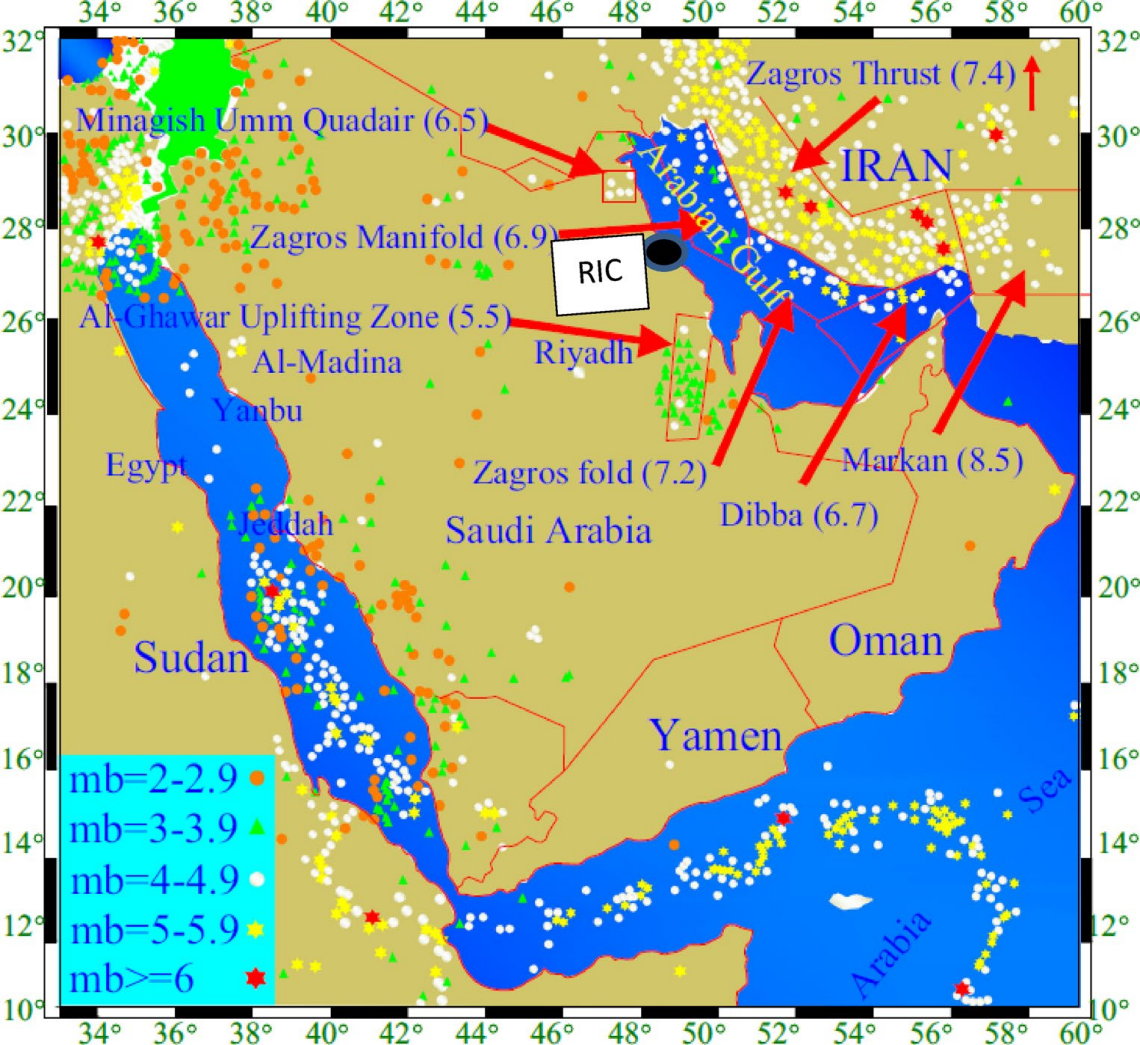
Seismic sources and distribution of seismic events in the vicinity of the study area (RIC)^22^.

**Fig. 5:**
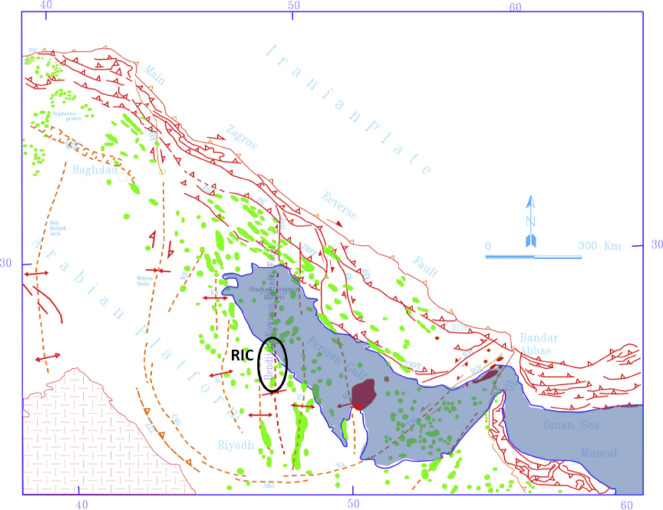
The Hendijane-Nowrooze-Khafji fault, in the vicinity of the study area (RIC)^38^.

The study area is located 250 km southwest of an active collisional plate boundary involving the Arabian and Eurasian plates, Fig. 3, which extends across the Zagros Mountains^5^. The central part of the Arabian Plate is moving northeastward^37^. This movement is coupled with a collision and subduction beneath the Eurasian plate, which causes the seismic events in the region. The main seismic/tectonic zones that may seismically affect the study area (RIC), in particular, are shown in Fig. 4, along with their associated maximum earthquake magnitudes^5^. There is a fault that extends from Eurasian plate to Arabian plate and pass close to the study area, the Hendijane-Nowrooze-Khafji fault, Fig. 5, ^38^. Seismotectonic analysis is reported in the literature^39^.

In the Zagros fold-fault Belt, earthquakes with a magnitude of 5 or greater frequently occur, while medium-sized earthquakes (magnitude 6+) happen several times annually. Additionally, large-magnitude earthquakes (7 and above) are recorded nearly every decade. Makran subduction zone, which forms the boundary between the Arabian and Eurasian Plates, is another potential source of major seismic events in the vicinity of the study area. This subduction zone can potentially generate severe seismic events like the Makran earthquake with a magnitude of 8.1 in November 1945^5^. A 7.8 magnitude earthquake shocked Iran in April 2013, and another one of magnitude 7.7 in September 2013^40^.

Other earthquakes that occurs close to the study area include ones in southwest Kuwait, with magnitudes of 4.7, 3.9, and 4.2 in 1993, 1997, and 1997, respectively. United Arab Emirates and Oman felt an earthquake of magnitude 5.2 in 2002^5^. A more recent event in Kuwait included an earthquake of magnitude 4.4 in 2022. These indicates the uncertainties nature in earthquake occurrences and magnitudes.

## Methodology

### Field and laboratory testing

In addition to the existing database, field and laboratory tests have been performed to acquire additional data, especially the dynamic parameters required for the 3-D modelling and simulations. These are presented in the following subsections. Table 2 provide the matrix for these field and laboratory tests. For quality assurance and control, these tests were conducted by certified testing labs. Procedures are certified as per the respective standard tests, staff are certified for qualification to conduct such tests, and equipment are certified for valid calibrations.

**Table 2:** Test matrix.

Test	Number
SPT (collected)	3765
Crosshole	3
Multichannel A S waves	3
Lab. cyclic triaxial	15
Resonant column	3
Direct shear	6
Permeability	6
SEM	4
ED XRF	4

#### Crosshole seismic test

Crosshole seismic tests were conducted at specified locations within each zone. They were performed according to the ASTM standard^41^. The standard penetration Tests (SPT) were also performed to determine N_60_ values, as per the ASTM standard^42^. This is done in each soil layer encountered during the drilling of the boreholes for the cross-hole tests. Furthermore, disturbed sand samples, undisturbed clay samples, and sabkha samples were collected during the borehole drilling. These were later used to prepare specimens for various laboratory tests. Samples of groundwater, including sabkha brine, were collected during borehole drilling.

#### Multichannel analysis of surface waves (MASW)

Multichannel analysis of surface waves (MASW) was carried out using an active method with a 24-channel seismograph, and a land streamer featuring receivers (geophones) with a frequency of 4.5 Hz. The seismic source consisted of a 90-N hammer and a steel plate. MASW was employed by multiple receivers (geophones) with an interval of 1.0 m along a linear survey line. MASW is being used for evaluation of dynamic properties of soils^43^, beside other techniques^44^.

#### Laboratory cyclic triaxial test

Laboratory cyclic triaxial tests were conducted on specimens of the soil samples collected during borehole drilling, as per the ASTM standard^45^. Specimens with a diameter of 50 mm and a height of 100 mm were prepared in five layers, following the compaction procedure outlined by^46^, to ensure consistent relative density throughout the specimen. To replicate the in-situ ground conditions, sample preparation involved using three types of fluid; sabkha brine, seawater, and normal groundwater, to determine the variation in seismic parameters associated with salt concentration in the pore fluid. Moreover, soil specimens were prepared at relative density levels of 40–85% and tested under confining pressures of 100–300 kPa. The chemical characterization of different types of waters was conducted using chemical analysis, and the results are provided in Table 3. A comprehensive range of dynamic parameters were obtained under various subsurface conditions and loading scenarios.

**Table 3:** Chemical characterization of different types of water.

Sample No	Sabkha Brine			Seawater			Normal Groundwater		
	Sample-1	Sample-2	Sample-3	Sample-1	Sample-2	Sample-3	Sample-1	Sample-2	Sample-3
Chlorides as Cl (PPM)	134,710	88,625	86,853	23,750	21,625	23,397	51	32	18
Sulphate as SO_4_ (PPM)	3119	17,644	4102	2832	3119	3242	17	12	8

*PPM* Parts per million.

#### Resonant column test

The resonant column tests were conducted according to the ASTM standard^47^. The test set-up consisted of a steel cell and a magnetic coil assembly designed to generate a sinusoidal torsional force at the top of the specimen. Additional equipment included a charge amplifier, an oscilloscope, a voltmeter, and a function generator. Sample preparation followed the same procedure that was described for the cyclic triaxial tests.

#### Direct shear test

Direct shear tests were performed to ascertain the changes in frictional resistance between sand particles in the presence of high saline brine in the sandy sabkha deposits. The tests were performed on the sand specimens saturated with normal water, and on the sandy sabkha specimens saturated with brine. The direct shear tests were carried out according to the ASTM^48^.

#### Permeability test

Permeability characteristics of the various forms of strata under study were carried out using falling/constant head permeameters. The specimens with and without brine were prepared in a Pyrex glass mold, and a water head was applied to the top of the specimens.

#### Scanning electron microscopy (SEM) and ED XRF

Four samples were prepared for SEM and ED XRF imaging and elemental analysis. Three samples are sand, saturated with normal water, seawater, and brine; respectively. The fourth sample is clay sabkha. Scanning Electron Microscope was used for SEM imaging, which provides ultra-high-resolution imaging at low accelerating voltages and small working distances. ED XRF analysis was carried out using a JEOL analyzer. The XRF outputs are quantifiable down to ppm levels. The irradiated area of a sample is much larger than that for the SEM.

### Modelling and simulations of seismic behavior

#### Modelling

In this study, the entire subsurface of the Ras Al Khair Industrial City (RIC) was modelled in PLAXIS 3D software. Based on the seismic and geotechnical aspects, the subsurface strata at RIC were divided into three zones, designated as sandy sabkha, clayey sabkha, and non-sabkha zones, as obtained from the lithology of the area, "Zonation of the site" section. So, three models were prepared in the software to represent these three zones. Each of these zones constituted several thicknesses of the various subsurface strata layers, and was confined by the respective boundary conditions. The Hadrukh formation, a hard clay, has been considered the bedrock for each zone.

**Fig. 6:**
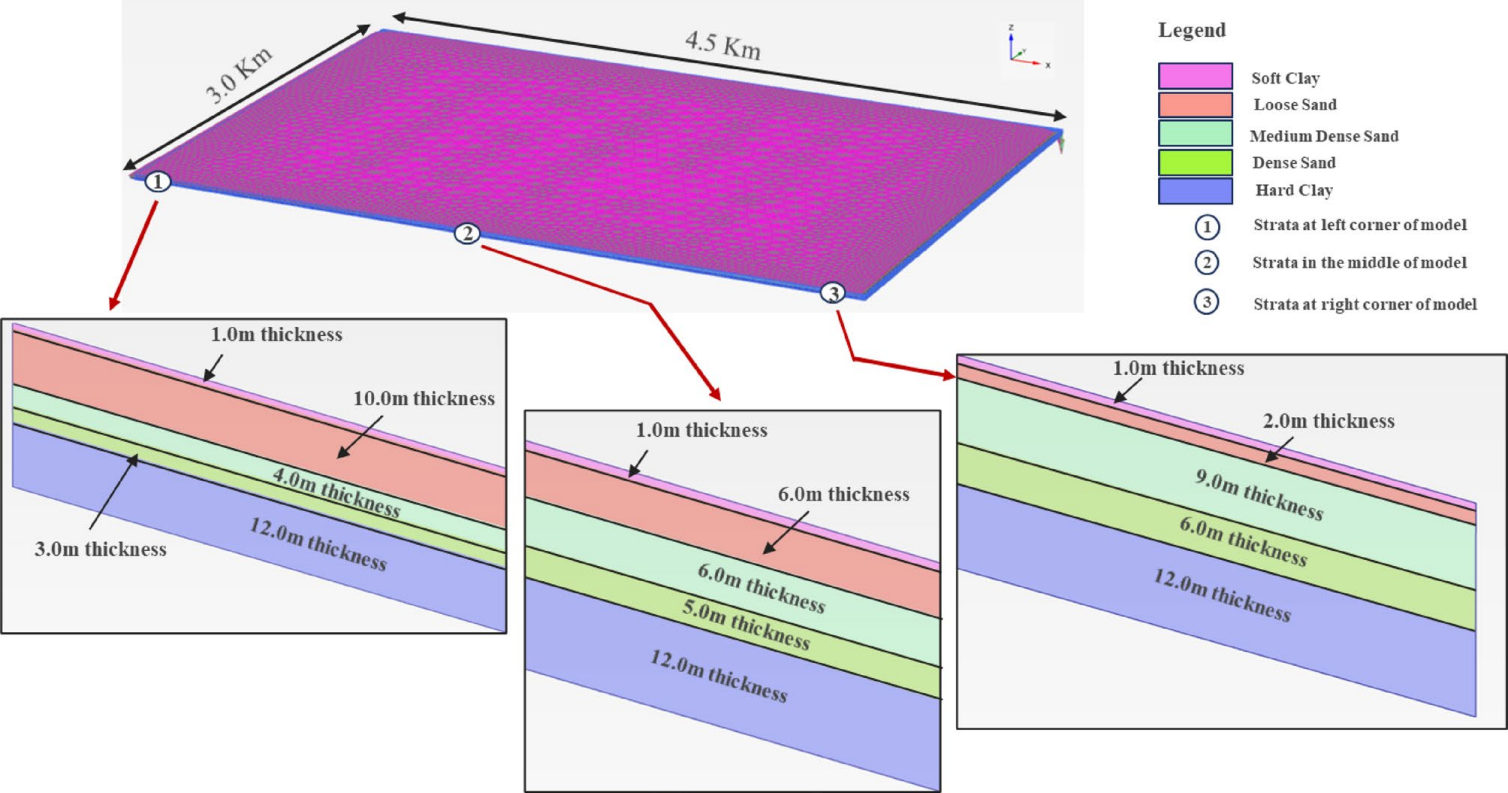
Plaxis 3D model for sandy sabkha zone.

The modelling was performed for each of the three zones with variations of lithological units. Fine mesh is used and the boundary conditions represents far-field. The sandy sabkha model is shown in Fig. 6, with the loose sand layer varying in thickness from 2.0 to 10.0 m. Similarly, the clayey sabkha model is constructed with the clay layer varying in thickness from 1.0 to 4.5 m. Also, the non-sabkha model constitutes medium-dense sand layers varying in thickness from 2.0 to 6.0 m. This is because the sandy strata are generally present in the medium-dense state from the ground surface to about of 6.0 m depth, the model for the non-sabkha zone.

**Fig. 7:**
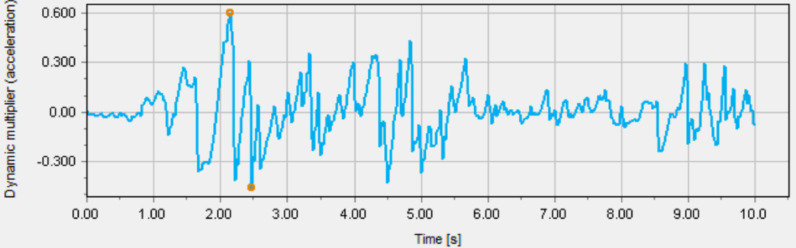
Proposed synthetic earthquake accelerogram for the Ras Al Khair Industrial City (RIC)^52^.

The layers in each zone were assigned static and dynamic parameters, as acquired from the field and laboratory tests discussed in "Field and laboratory testing" section. For a typical site-specific seismic simulation, an earthquake was also applied at the bedrock as peak ground acceleration (PGA). The local seismic design codes^49–51^, were used to select the input earthquake parameters, Table 4. The seismic design acceleration value of 0.06 g, as specified in the Saudi building code, is more conservative and has been adopted as the design acceleration. In the absence of any past earthquake accelerogram of the region, a synthetic earthquake was adopted for seismic simulation. It is adopted from the earthquake catalogue of Saudi Geological Survey^52^ for a10-sec duration and maximum PGA of 0.060 g, Fig. 7.

**Table 4:** Seismic parameters from local design codes.

Code/Standard/Reference	Short period (0.2 s) Acceleration, S_s_ (% g)	1 s period Acceleration, S_1_ (% g)	PGA (% g)
Saudi Building Code SBC 301 (2018)^51^	11.0	7.0	6.0
Royal Commission Engineering Manual (2022)^50^	10	4.67	4.0
Saudi Aramco SAES-A-112 (2022)^49^	8.5	3.1	3.4

#### Simulations

The dynamic response of each of the three zones was performed using Plaxis 3D software^53^. The hardening soil with small-strain stiffness (HSS) model was used for dynamic analysis due to its consideration of hysteresis in cyclic loading and corresponding damping^54^. The HSS constitutive model consists of 13 parameters related to strength, stiffness, and strain. The most critical parameters for dynamic analysis are small-strain shear modulus (G_o_ or G_max_) and the shear strain level (γ_0.7_). These were obtained from the field and laboratory tests, as explained in "Field and laboratory testing" section. The strength parameters (effective cohesion, c′; effective angle of internal friction, φ′; failure ratio, R_f;_ and reference secant modulus, E_50_^ref^) were obtained from available results of the consolidated drained triaxial test, conducted as per ASTM standard^55^. The other model parameters are derived using empirical relationships: E_oed_^ref^ = 0.89 Es, E_50_^ref^ = 1.13 E_oed_^ref^, E_ur_^ref^ = 3.89 E_50_^ref^, and G_max_ = 2.0 E_ur_^ref^, suggested by^56^. The design input parameters for HSS model for all zones were compiled. The parameters for sandy sabkha zone are provided in Table 5, where $\upgamma$_0.7_ is the threshold shear strain at which G_max_ = 0.722G_0_, E_ur_^ref^ is the unloading/reloading stiffness from the drained triaxial test, E_50_^ref^ is secant stiffness in the drained triaxial test, E_oed_^ref^ is the tangent stiffness from the primary oedometer loading, υ_ur_ is the elastic Poisson’s ratio, m is the power for stress-level dependency of stiffness, and D is the damping ratio.

**Table 5:** The input parameters of HSS model for the sandy sabkha zone.

Soil Type	Design G_max_ (MPa)	Design $\upgamma$_0.7_	E_ur_^ref^ (MPa)	E_50_^ref^ (MPa)	E_oed_^ref^ (MPa)	υ_ur_	m	D (%)
Sabkha (clay)	4.0	1.205E−03	2.0	0.5	0.5	0.45	1.00	5.00
Sabkha (sand)	9.0	7.217E−04	4.5	1.2	1.0	0.30	1.00	3.00
Medium dense sand	177.0	8.832E−05	88.5	22.8	20.1	0.30	0.80	2.00
Dense sand	397.0	6.208E−05	198.5	51.0	45.2	0.30	0.70	2.00
Hard clay	512.0	1.736E−04	256.0	65.8	58.2	0.35	0.65	3.00

### Liquefaction potential

The liquefaction potential of all each of the three zones under seismic loading was assessed; using the UBC3D-PLM model in the software^57,58^, which is an extension of the UBCSAND model proposed by^59,60^. The UBC3D-PLM model consists of 11 parameters, which were obtained from equations based on SPT (N_1_)_60_ values^61^. The SPT profiles (Fig. 2) were used to obtain these parameters. Several earthquake shakings (0.060 to 0.1 g) were imposed on the models, to examine the liquefaction potential at different earthquake magnitudes. The design input parameters for UBC3D-PLM model for all zones were compiled. The parameters pertaining to the sandy sabkha zone are provided in Table 6, where N_60_ & (N_1_)_60_ are the corrected SPT-N values, k^*e^_B_ is the elastic bulk modulus factor, k^*e^_G_ is the elastic shear modulus factor, k^*e^_G_ is the plastic shear modulus factor, m_e_ or n_**e**_ is the rate of stress-dependency of elastic bulk modulus = 0.5 for all soils, n_p_ is the rate of stress-dependency of plastic shear the modulus = 0.4 for all soils, p_ref_ is the reference pressure = 100 kPa, φ_cv_ is the constant volume friction angle, φ_p_ is the peak friction angle, c is the cohesion, σ_t_ is the tension cut-off and tensile strength = 0 for all soils, R_f_ is the failure ratio, f_dense_ is the densification factor = 1 for all soils, and f_Epost_ is the post-liquefaction factor.

**Table 6:** The design input parameters for UBC3D-PLM model for the sandy sabkha.

Soil type	N60	(N_1_)_60_	k*^e^_B_	k*^e^_G_	k*^p^_G_	φ_cv_ (deg)	φ_p_ (deg)	C (KPa)	R_f_	f_Epost_
Sabkha (clay)	2	2	383	547	107	28	28	10	0.9	0.2
Sabkha (sand)	3	4	497	710	141	30	30	0	0.88	0.2
Sand	20	20	819	1169	1444	33	36	0	0.70	0.5
Sand	49	39	1031	1474	6876	35	44	0	0.63	1.0
Clay	100	100	1410	2014	60,524	1	28	200	0.55	1.0

**Fig. 8:**
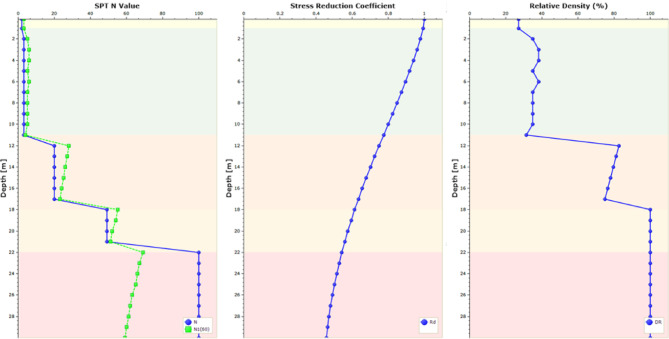
Settle3D model for sandy sabkha zone.

To verify the results obtained from the above simulations, the liquefaction potential was also determined using Settle3D software^62^. Settle3D utilizes several empirical methods for liquefaction analysis^63–66^. The input parameters for this software are the SPT (N_1_)_60_ values, PGA, and the earthquake magnitude. The input parameters for the software are provided in Fig. 8, in addition to considering an earthquake magnitude of 6.5.

## Results and discussions

### Zoning map of the subsurface strata

**Fig. 9:**
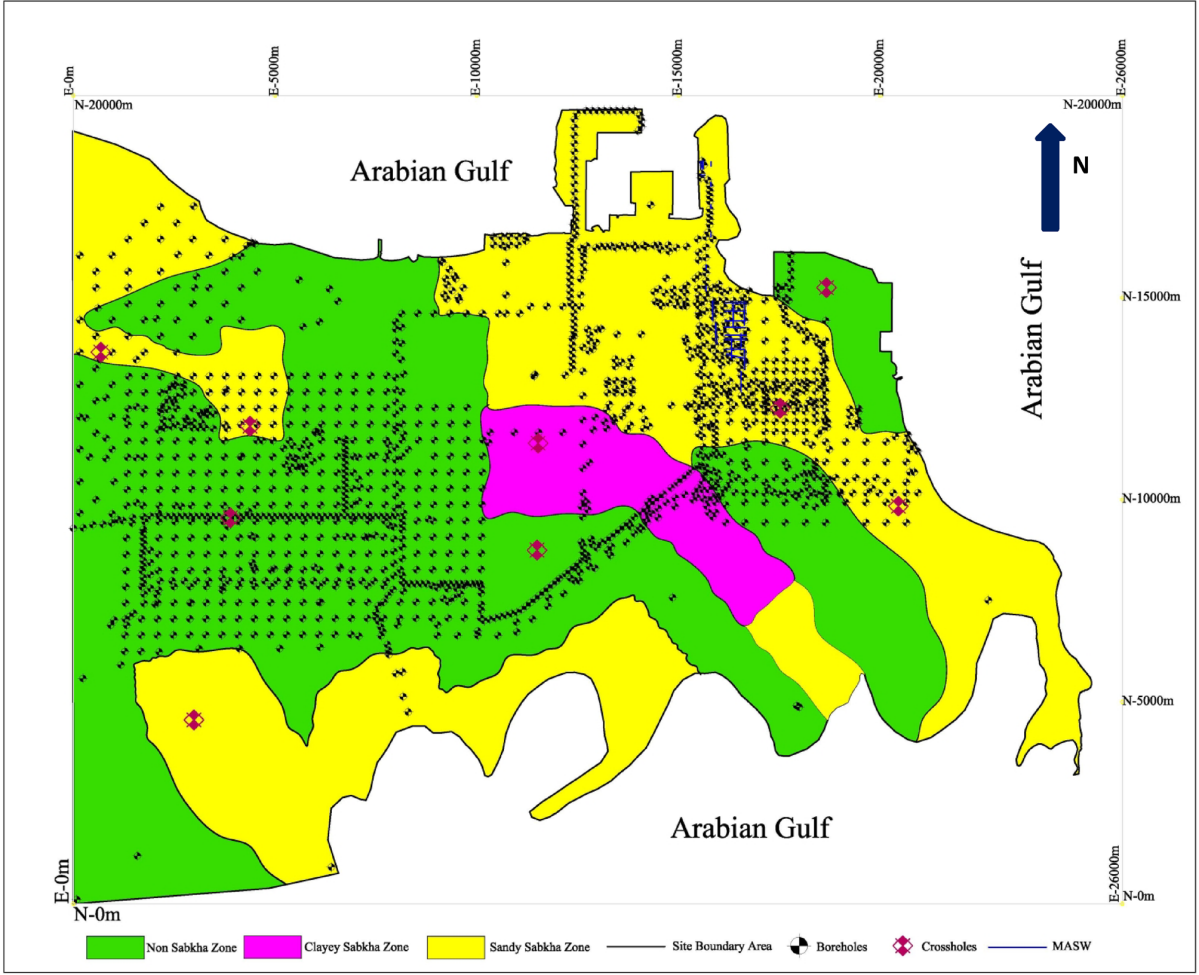
Subsurface strata zoning map of the study area.

Based on the lithological units shown in Fig. 2, the zoning map for the subsurface strata of the site is presented in Fig. 9. It shows the three distinct zones, sandy sabkha, clayey sabkha, and non-sabkha zones. Also, it indicates the locations for the field tests, the SPT boreholes, the cross hole, and the MASW.

### Field and laboratory results

**Fig. 10:**
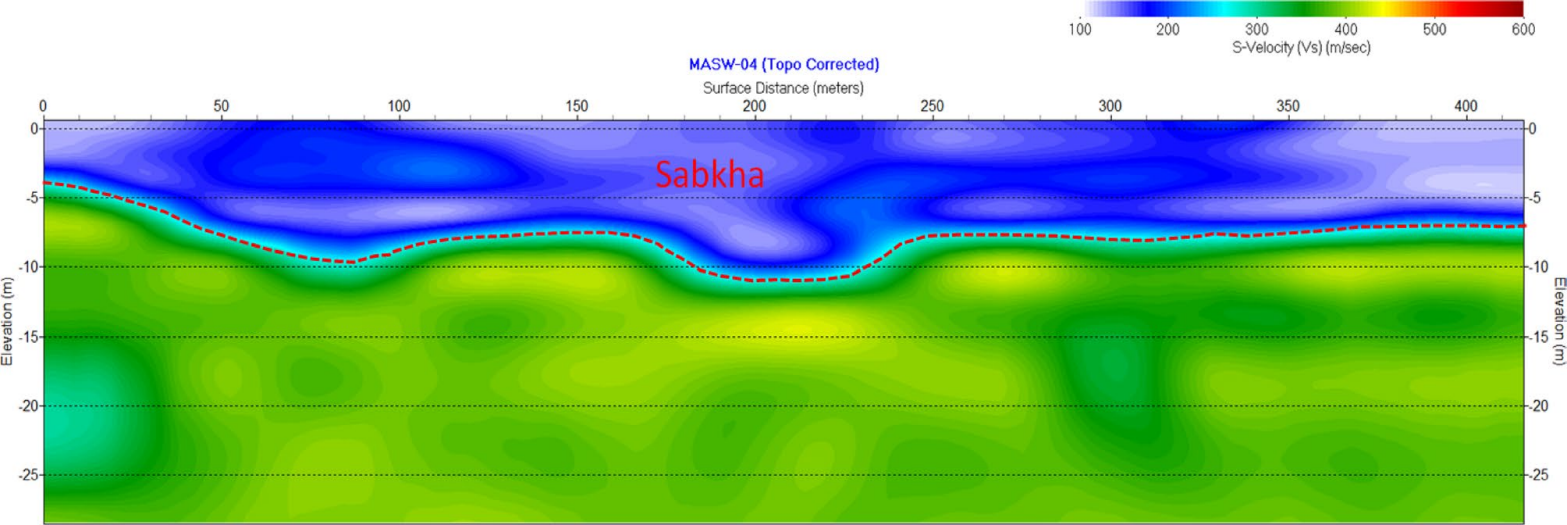
MASW results showing the variation of shear wave velocity (V_S_) with depth in the sandy sabkha zone.

The seismic filed tests include crosshole tests and MASW tests. The results of the crosshole tests to a standard depth on 30 m were compiled for all zones. Table 7 shows a typical subsurface profile for sandy sabkha zone. On the other hand, Fig. 10 shows a typical MASW section obtained for sandy sabkha zone. The acquired seismic parameters are summarized and presented in Table 8.

**Table 7:** Crosshole test results for the sandy sabkha zone.

Depth of source & receiver (m)	Distance from source to R1 (m)	Travel time of the wave from source to R1 (msec)		Distance from source to R2 (m)	Travel time of the wave from S to R2 (msec)		Average wave velocity (m/sec)		Density ρ (g/cm^3^)	Poisson Ratio υ	Shear Modulus G (MPa)
		P	S		P	S	P	S			
0.75	3.00	23.93	58.01	6.00	55.87	143.82	116	47	1.35	0.404	2.9
1.50	3.00	15.10	43.68	6.00	33.21	113.88	190	61	1.35	0.443	5.0
3.00	3.00	22.27	47.43	6.00	48.89	132.59	129	54	1.45	0.392	4.0
4.50	3.00	19.34	36.15	6.00	42.50	84.51	148	77	1.49	0.315	9.0
6.00	3.00	18.74	38.84	6.00	41.63	101.26	152	68	1.48	0.374	7.0
7.50	3.00	16.86	32.07	6.00	35.73	75.43	173	87	1.47	0.333	11.0
9.00	3.00	18.41	34.66	6.00	41.39	85.03	154	79	1.45	0.324	9.0
10.50	3.00	21.14	45.88	6.00	50.87	112.39	130	59	1.43	0.368	5.0
12.00	3.00	21.65	41.50	6.00	50.59	119.29	129	61	1.40	0.353	5.0
12.75	3.00	3.54	7.81	6.00	7.20	16.20	840	377	1.67	0.374	238.0
15.00	3.00	3.69	7.26	6.00	7.58	15.25	802	403	1.65	0.331	268.0
18.00	3.00	4.50	8.31	6.00	9.28	17.64	657	351	1.57	0.301	193.0
21.00	3.00	2.29	6.53	6.00	4.65	13.76	1300	448	1.86	0.433	373.0
24.00	3.00	2.78	7.75	6.00	5.68	16.59	1068	374	1.77	0.430	248.0
27.00	3.00	2.93	7.91	6.00	6.00	17.04	1012	366	1.75	0.425	234.0
30.00	3.00	2.96	6.76	6.00	6.07	14.48	1001	429	1.74	0.387	321.0

R1 = First receiver hole; R2 = Second receiver hole; P = Primary wave; S = Secondary wave.

**Table 8:** Summary of seismic parameters acquired from MASW in the sandy sabkha zone.

Depth (m)	Type of material	Average shear wave velocity V_S_ (m/sec)	Density ρ (g/cm^3^)	Poisson ratio υ	Shear modulus G (MPa)
0–12	Sabkha (sand)	190	1.40	0.40	51
12–18	Sand (medium dense)	323	1.70	0.33	177
18–22	Sand (dense)	457	1.90	0.33	397
22–30	Clay (hard)	506	2.00	0.40	512

**Fig. 11:**
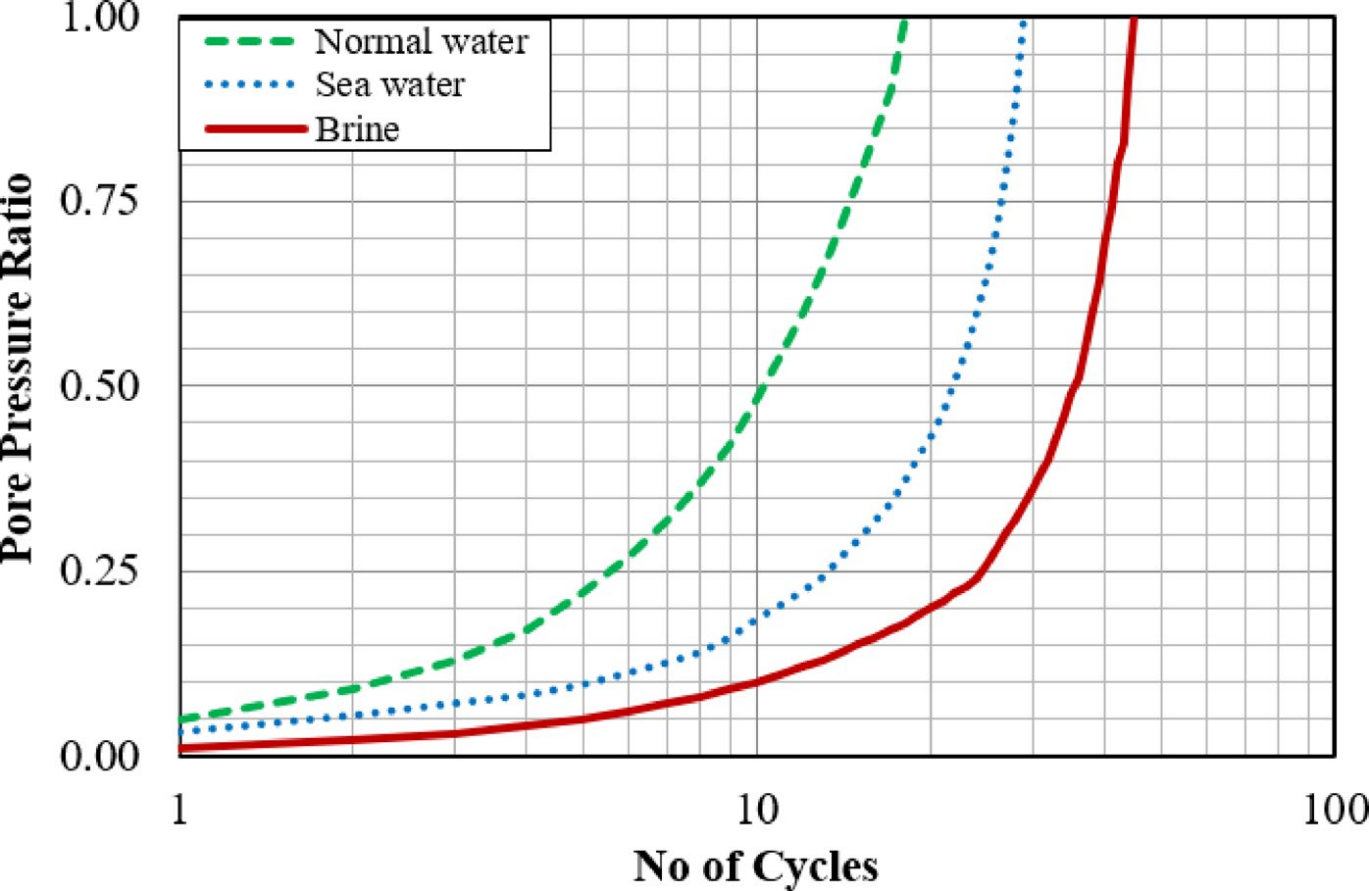
Typical results for the sand specimen at D_r_ = 85%, stress ratio = 0.4, frequency = 1 Hz, and confining stress = 300 kPa.

**Fig. 12:**
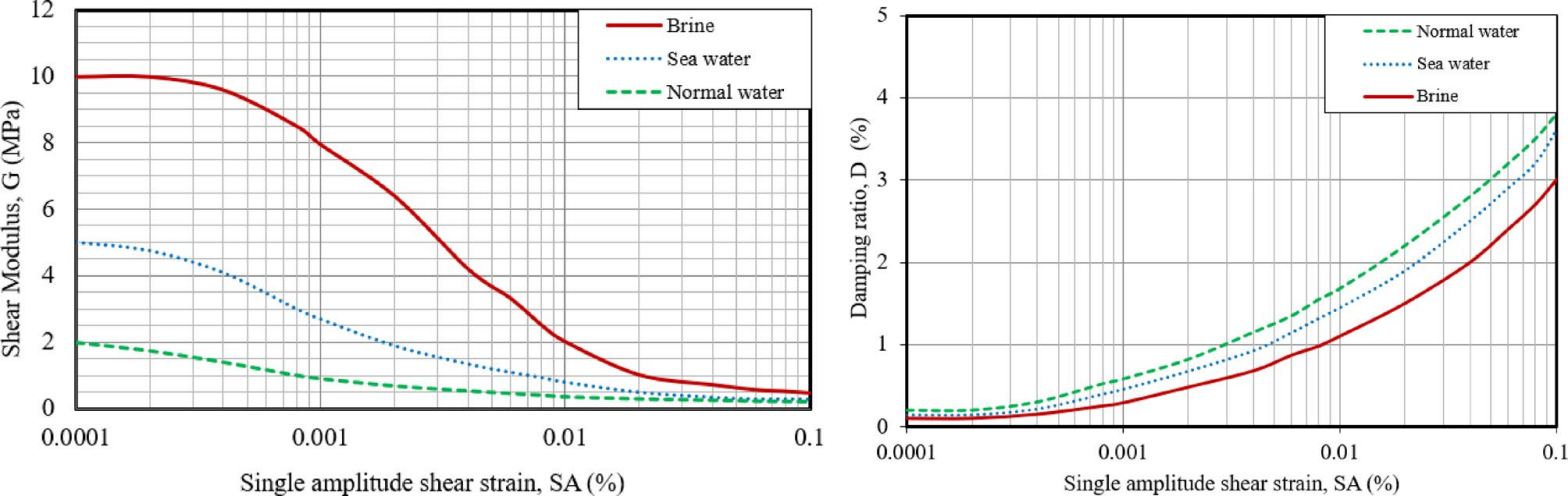
Resonant column test results showing the variation of shear modulus (G) and damping ratio (D) of the sand sample.

**Fig. 13:**
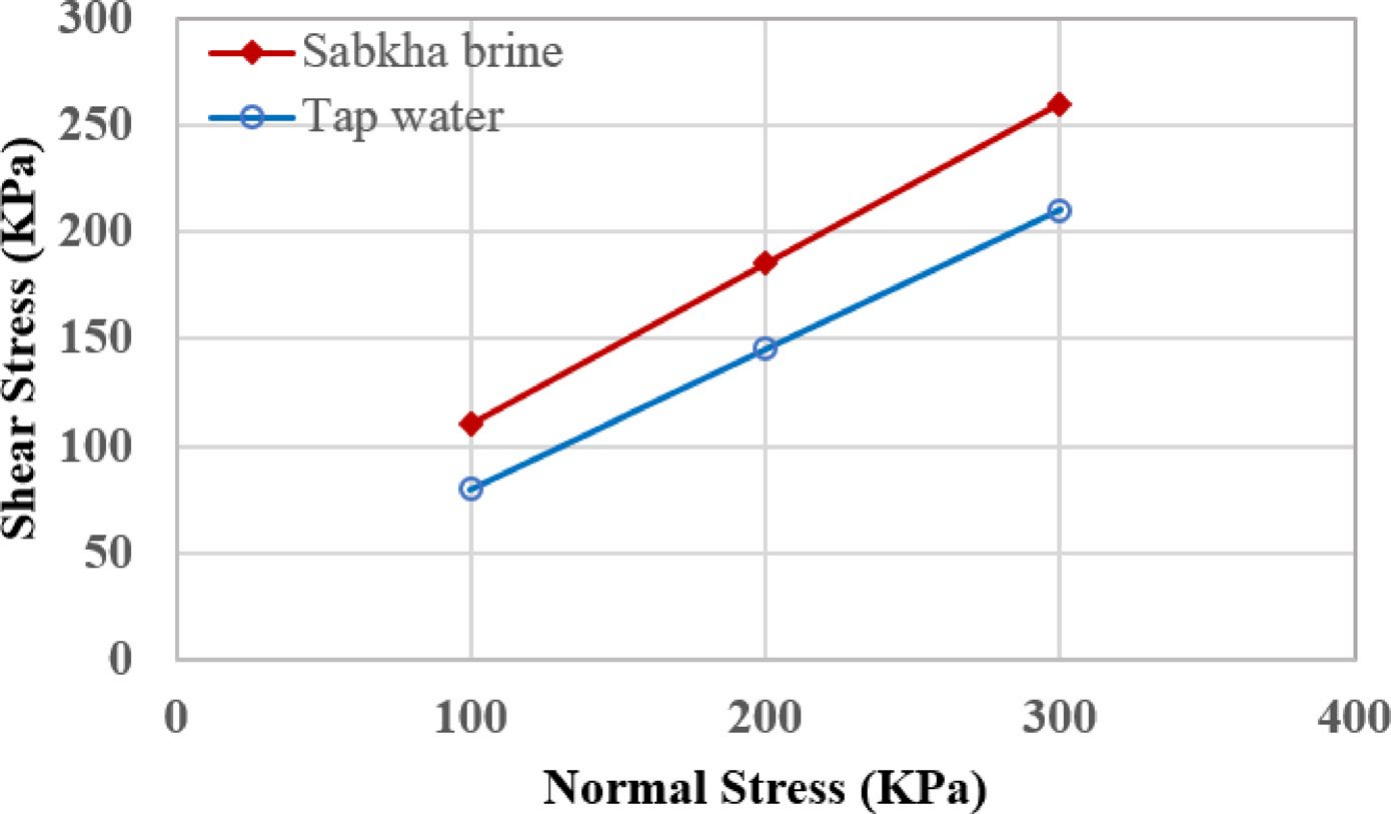
Results of the direct shear tests on sand specimens saturated with tap water and with brine.

The laboratory strength tests include cyclic triaxial tests, resonant column tests, and direct shear tests. Figure 11 presents typical results from the cyclic triaxial tests on sand samples, for pore-pressure ratio versus number of cycles. The test results are summarized in Tables 9 and 10 in terms of young’s modulus and axial damping ratio. Also, typical results obtained from resonant column tests on sand samples are illustrated in Fig. 12. The results are summarized in Table 11, in terms of the Young’s modulus and the axial damping ratio (λ). It is evident from the results that the samples saturated with brine exhibit a significantly higher stiffness and lower damping, compared to those with normal groundwater and seawater. This phenomenon, most probably, is owed to the much higher viscosity/density of the sabkha brine. The effect of high salt concentrations in the pore water (brine) on the dynamic response of soils can be established from the increase in the shear modulus of soils containing seawater. Furthermore, the results of the direct shear tests on sand samples are shown in Fig. 13. The results reveal an angle of internal friction ($\upphi$) increase from 33° for the one with normal/tap water to 37° for the specimen with brine fluid. This anomalous behavior is attributed to the additional frictional resistance due to the salt precipitated on the grains of the sandy sabkha soils.

**Table 9:** Young’s modulus (MPa) from the cyclic triaxial tests.

Relative density (%)	Confining pressure, $\upsigma$_3_ (KPa)	(Stress ratios/frequency)										
		(.025/.1)	(.05/.1)	(.075/.1)	(.100/.1)	(.125/.1)	(.150/.1)	(.025/1)	(.05/1)	(.075/1)	.100/1	.100/1
40	100	14	7	5	–	–	–	9	6	4	–	–
60	150	54	38	24	11	–	–	39	30	18	–	–
75	200	143	100	55	28	–	–	87	57	46	27	–
85	300	286	240	180	80	47	20	158	109	75	48	21

**Table 10:** Axial damping ratio, λ (%), from the cyclic triaxial tests.

Relative density (%)	Confining pressure, $\upsigma$_3_ (KPa)	(Stress ratios/frequency)										
		(.025/.1)	(.05/.1)	(.075/.1)	(.100/.1)	(.125/.1)	(.150/.1)	(.025/1)	(.05/1)	(.075/1)	.100/1	.100/1
40	100	6.39	10.43	15.90	–	–	–	9.61	14.24	19.25	–	–
60	150	5.23	8.11	11.83	15.97	–	–	6.54	9.72	13.57	–	–
75	200	3.79	7.19	11.83	14.28	–	–	5.95	9.69	12.91	16.55	–
85	300	4.17	7.18	11.92	14.95	16.42	19.16	5.89	9.16	13.91	16.86	19.49

**Table 11:** Summary of resonant column test results.

Type of soil	Relative density (%)	Confining pressure, $\upsigma$_3_ (KPa)	Maximum shear modulus, G_max_ (MPa)	Damping ratio, D (%)	γ_0.7_
Loose sand sabkha (with brine)	40	100	10	3.0	7.217 × 10^–4^
Loose sand sabkha (with sea water)	40	100	5	3.6	7.217 × 10^–4^
Loose sand sabkha (with normal water)	40	100	2	3.8	7.217 × 10^–4^
Medium dense sand	60	150	200	2.0	4.402 × 10^–5^ to 8.832 × 10^–5^
Dense sand	75	200	320	2.0	6.208 × 10^–5^
Very dense sand	85	300	425	2.0	8.03 × 10^–5^

**Fig. 14:**
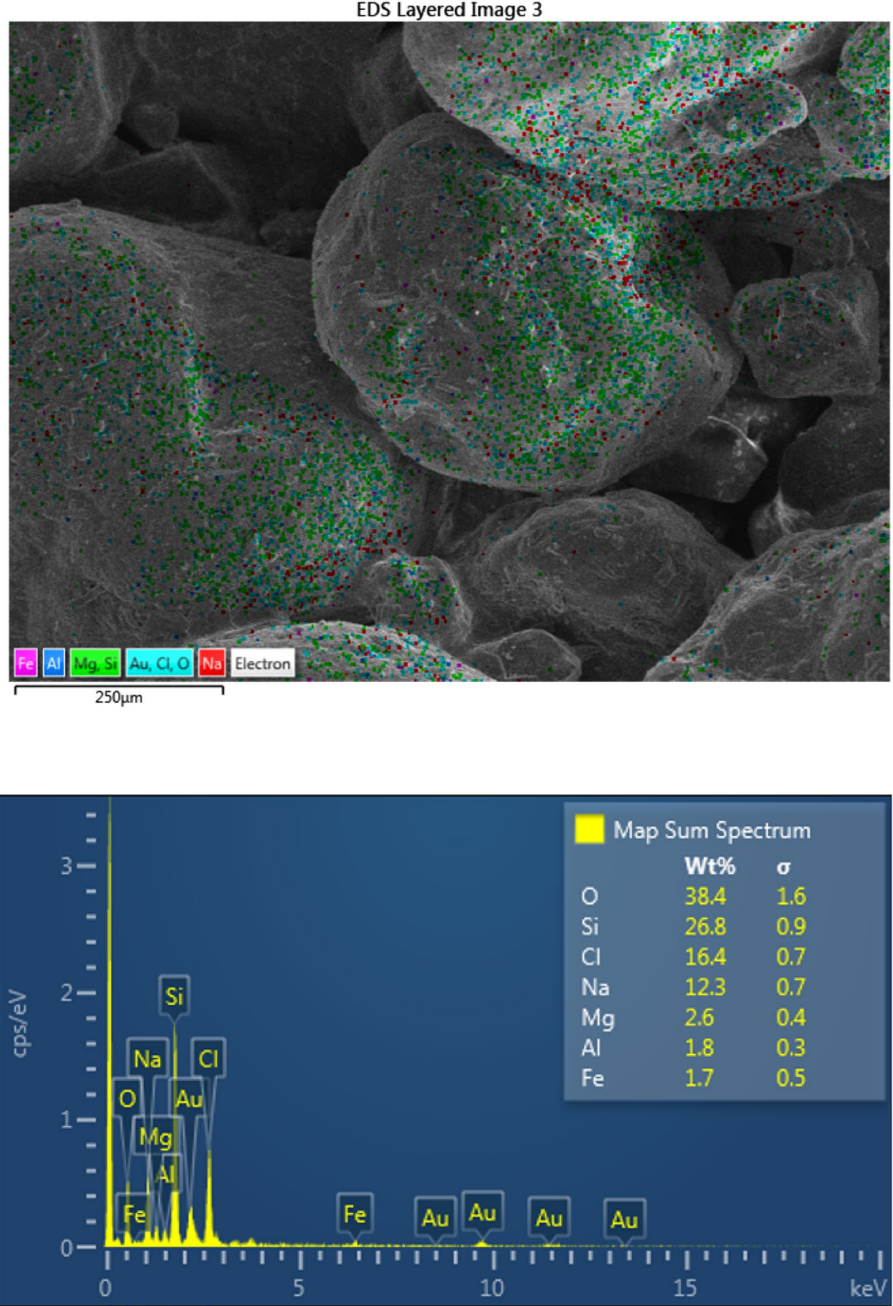
(**a**) SEM images of sandy sabkha sample with brine, at different magnifications. (**b**) ED XRF image and element analysis of sandy sabkha sample with brine.

Other laboratory tests include permeability tests, SEM, and ED XRF. The values for the coefficient of hydraulic permeability were determined as a result of the permeability tests, as listed in Table 12. Additionally, the results of the SEM and ED XRF analysis and imaging were compiled for all four samples. The results pertaining to sand sample with brine are presented in Fig. 14a,b, from SEM and ED XRF, respectively. The elemental analysis indicates that this sample contains NaCl of 16.44% by weight, which confirms high salt content in sandy sabkha.

**Table 12:** Coefficient of hydraulic permeability (k) of various soils for different types of water.

Soil	Water type	k (m/s)
Sand	Regular water	3.5 × 10^–5^
Sand	Seawater	2.5 × 10^–6^
Sand	Brine	6.0 × 10^–8^
Clayey sabkha		5.0 × 10^–12^

### Simulation of seismic behavior

The results from the numerical simulations of the seismic behavior are presented in the following sections, as attenuation response, liquefaction response, and spectral response. The anomalous behaviors and responses are also discussed.

#### Attenuation response

**Fig. 15:**
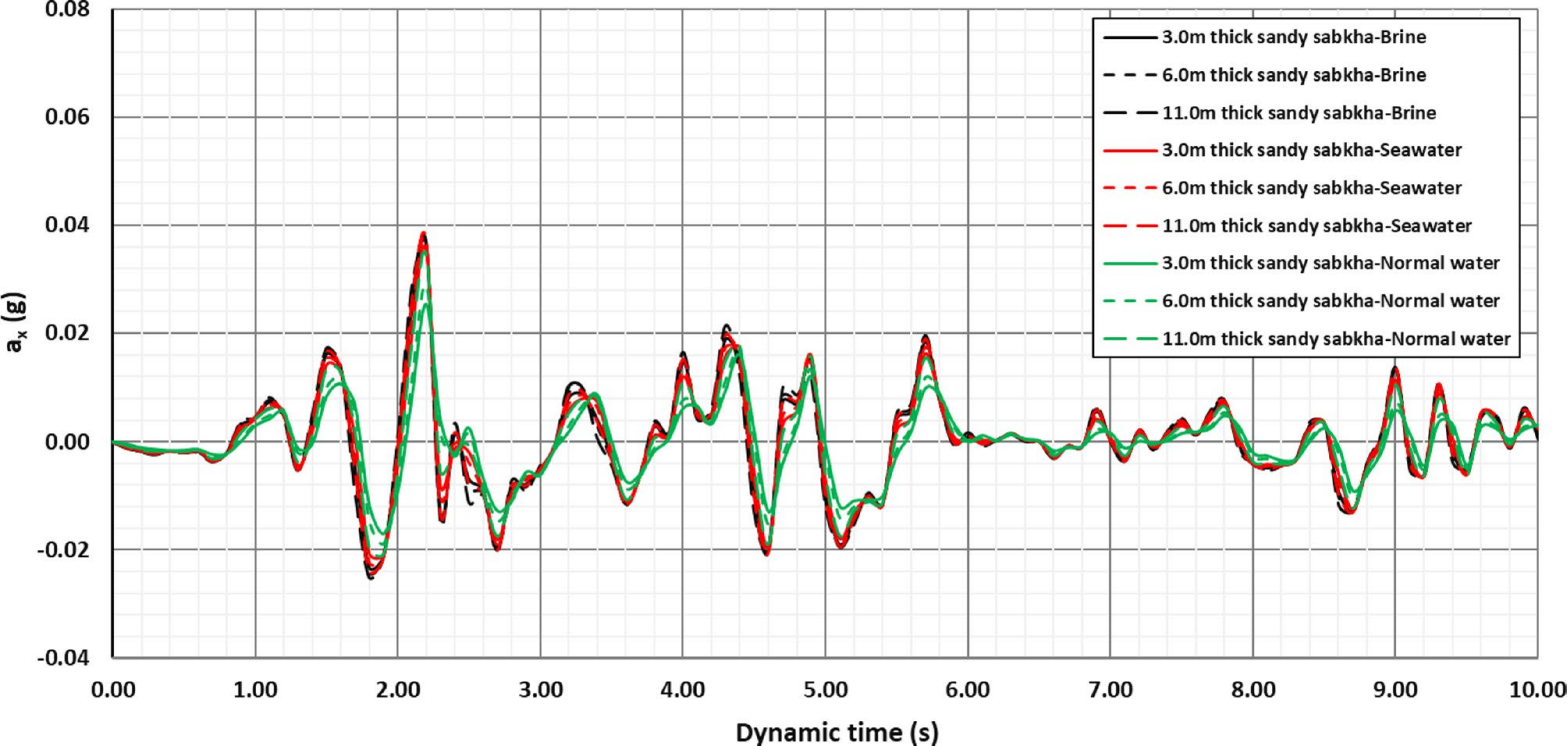
Acceleration vs time history for the sandy sabkha zone.

The acceleration versus time plots were made for the three zones. The record for sandy sabkha zone is presented in Fig. 15. This reveals a tendency towards attenuation of the seismic acceleration as the waves propagate from the bedrock to the ground surface. The seismic acceleration of 0.06 g at the bedrock attenuates to 0.03–0.04 g at the ground surface for the different zones. The attenuation is maximum for normal groundwater as the pore fluid, while it is relatively lower in the case of brine-saturated sabkhas (Fig. 15). This agrees with the damping ratio presented in Fig. 12, that the higher damping ratio is associated with the normal groundwater. These are controlled by the wave dispersion characteristics of the soil media, in which different frequency components of a seismic wave travel at varying velocities. This causes the wave to spread out as it propagates through the soil. Seismic wave dispersion is influenced by several factors, including soil properties (type, void ratio, moisture content), frequency, and the complex interactions within the soil structure.

The presence of pores in the soil, as well as the nature and type of pore fluid, play a significant role in determining the extent of the dispersion of seismic waves. The attenuation is generally controlled by the resistance of the soil structure to the wave propagation. Therefore, the low attenuation in sabkha soils can be ascribed to the presence of brine in the pores, and the rigid structure created by the salts precipitated on the soil grains. The pore water of high salt content has higher viscosity, that permits the wave to propagate with less attenuation. The soil structure for the sandy sabkha, shown in Fig. 14a, reveals that salt precipitation on sand grains of sabkha soils. High salt concentration, as evidenced in Fig. 14b, also confirms the presence of the precipitated salt. The relatively higher rigidity of the brine-saturated sabkha is also evident from the shear modulus values determined in MASW and resonant column test results (Fig. 10 and Tables 8 and 11). In contrast, the low viscosity of normal water and the relatively less rigid soil structure, resulted in higher attenuation^67,68^. It can be established that the 3-D models result in a more realistic geometric damping, or attenuation response, than in strata than the 1-D analysis, as have also been reported by^69,70^. This is further established through the results of direct shear tests, where the frictional resistance for the sabkha samples is much higher than that of those saturated with regular water (Fig. 13). The increase in frictional resistance can be attributed to the presence of a high concentration of salt crystals in the pore of sabkha soils.

In addition to the contribution of the high salt content to the material damping that affects the attenuation, geometric attenuation is also anticipated due to the use of 3-D modeling in the numerical simulation. Another established concept, reported in the literature^23^, is the amplification of seismic waves in similar strata when the soil strata are modelled as a 1-D column. However, in this current study, an attenuation has been observed instead of amplification. This fact has also been reported in the literature^28^ and is most likely due to the wave dispersion effect in the 3-D. This attenuation, resulting from wave dispersion effects in a 3-D model, has led to a more realistic model. Therefore, both material and geometric damping contribute to the anomalous soil attenuation for different cases explored in this current study.

#### Spectral response

**Fig. 16:**
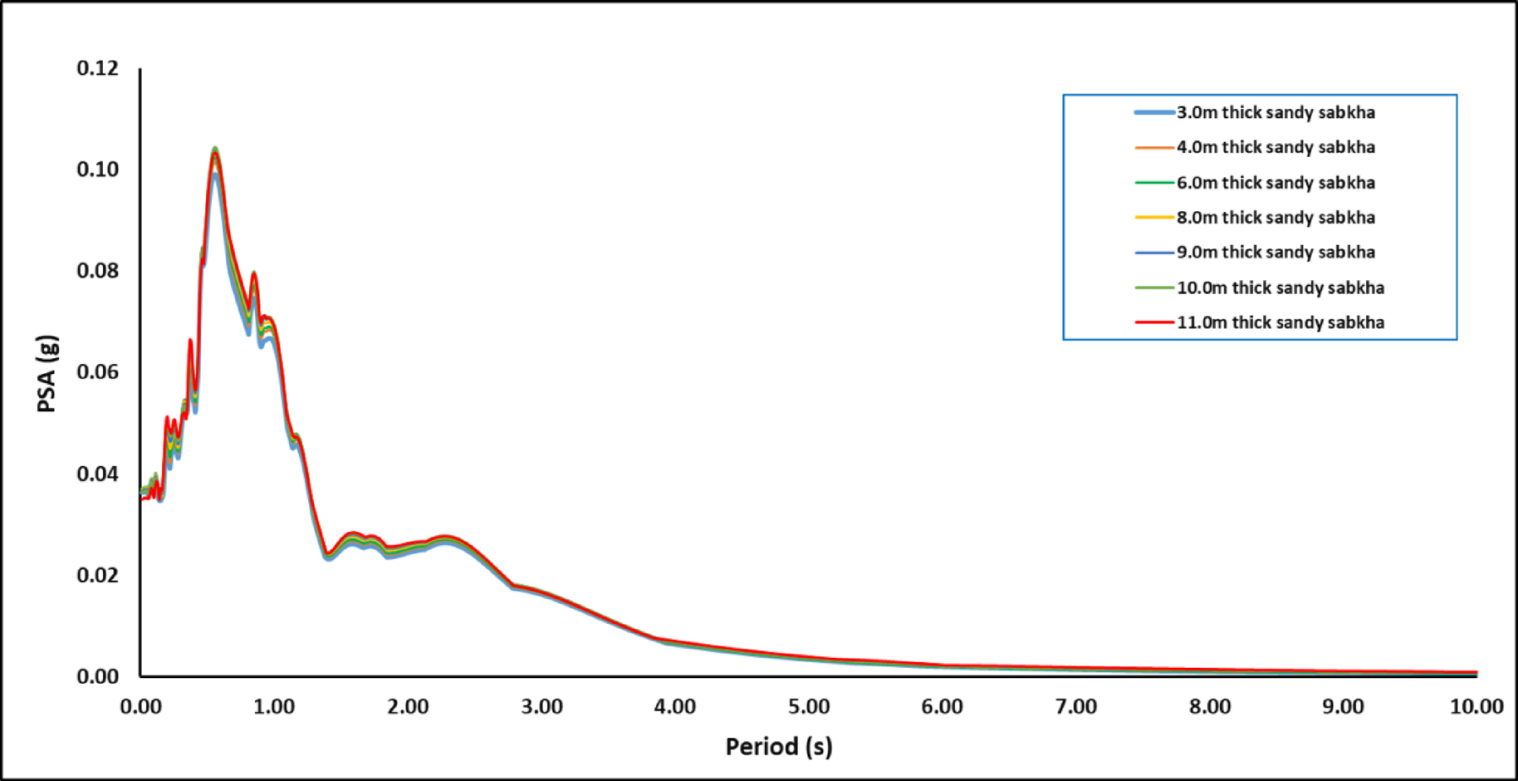
PSA variation in the sandy-sabkha zone with brine.

**Fig. 17:**
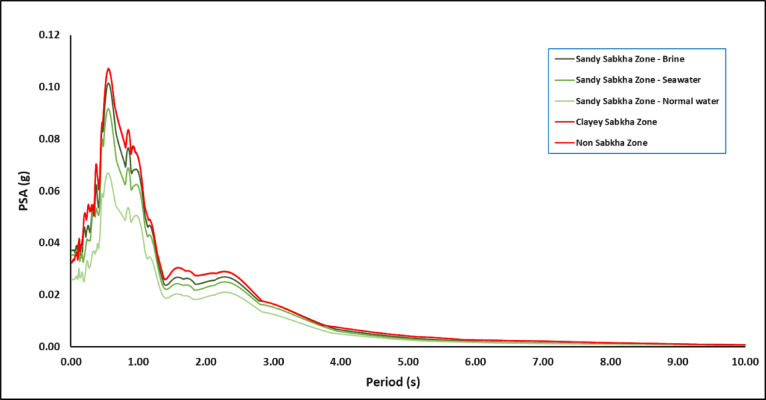
PSA variation in all zones.

Figures 16 presents a typical output of the seismic response spectrum for the sandy sabkha zone, with a 5% damping ratio for the structure. Also, Fig. 17 presents spectra for all zones. The site-specific spectral acceleration responses for all zones are summarized in Table 13. Notice that the values of the spectral accelerations (S_s_ and S_1_) obtained in this study are different than those ones specified by local codes (Table 4). The S_s_ values acquired from the simulation results range from 0.026 to 0.051 g, which are lower than those specified by the codes (0.09–0.15 g). In contrast, S_1_ values vary from 0.05 to 0.072 g, which are higher than those specified by the codes (0.03–0.07 g). This difference in S_s_ values is attributed to the differences between 3-D analysis used in the current study and the 1-D analysis adopted by the codes. Moreover, the 1-D equivalent analysis carried out in a previous study by^22^ in a close-by area confirms this difference. This shows the importance of the 3-D analysis to obtain spectral accelerations that are more representative of the ground behaviour.

**Table 13:** Summary of spectral acceleration responses for the three zones.

Zone	a_max_/g	Sub-zone Criteria		Modeling Results						Values from Codes								
		Thickness of loose/soft layer of sabkha (m)	Thickness of medium dense sand (m)	S_S_			S_1_			S_S_			S_1_			PGA		
				Normal water	Sea water	Brine	Normal water	Sea water	Brine	SBC 301	RC GEM	SAES-A-112	SBC 301	RC GEM	SAES-A-112	SBC 301	RC GEM	SAES-A-112
Sandy sabkha	0.06	3.0	–	0.0261	0.0352	0.0450	0.0497	0.0616	0.0656	0.15	0.1	0.085	0.07	0.047	0.031	0.06	0.04	0.034
		4.0	–	0.0278	0.0362	0.0461	0.0522	0.0634	0.0672									
		6.0	–	0.0292	0.0379	0.0472	0.0540	0.0643	0.0677									
		8.0	–	0.0311	0.0407	0.0487	0.0564	0.0654	0.0684									
		9.0	–	0.0328	0.0435	0.0498	0.0587	0.0664	0.0691									
		10.0	–	0.0345	0.0459	0.0507	0.0607	0.0672	0.0694									
		11.0	–	0.0355	0.0485	0.0513	0.0620	0.0671	0.0689									
Clayey sabkha	0.06	1.0	–	––	–	0.0507	–	–	0.0699	0.15	0.1	0.085	0.07	0.047	0.031	0.06	0.04	0.034
		1.5	–	–	–	0.0487	–	–	0.0699									
		2.0	–	–	–	0.0510	–	–	0.0723									
		2.5	–	–	–	0.0510	–	–	0.0723									
		3.0	–	–	–	0.0510	–	–	0.0723									
		4.0	–	–	–	0.0505	–	–	0.0707									
		4.5	–	–	–	0.0481	–	–	0.0672									
Non-sabkha	0.06	–	2 to 6	–	–	0.0510	–	–	0.0730	0.15	0.10	0.09	0.07	0.05	0.03	0.06	0.04	0.03

Moreover, it appears that the S_s_ values are more affected by the dispersion of the waves in 3-D. The S_1_ remain unchanged for both 3-D and 1-D modelling. The fact that the S_s_ is more affected and the S_l_ is least affected by the 3-D dispersion could be attributed to the presence of a sedimentary basin in the study area, as concluded by^29,30^. Although the S_1_ values for sabkha layers are close to those specified in the Saudi Building Code, the code provides only a single value of 0.07. This study reveals a range of S_1_ values (0.066–0.073) for different zones. This difference is attributed to a 3-D wave dispersion effect, considered in the present study, as compared to the 1-D analysis generally conducted for the seismic code development. This is one of the advantages of performing 3-D seismic response analysis, which considers the effects of local variations in geology, type, nature of subsurface strata, topography, and boundaries of various local lithological units. The 3-D modelling and simulations leads to a more realistic representation of ground conditions.

#### Liquefaction response

**Fig. 18:**
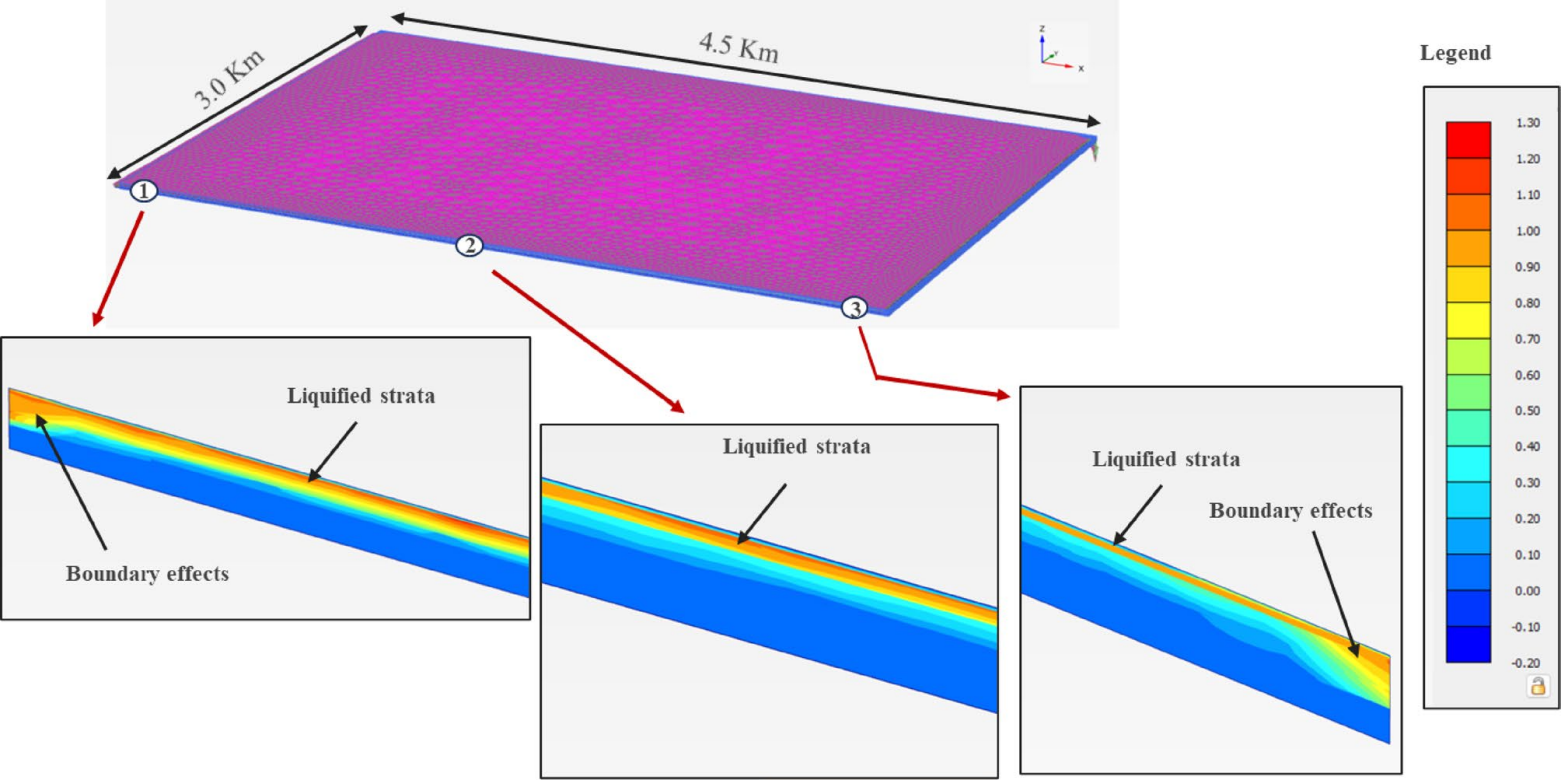
Liquefaction potential of sandy sabkha zone at PGA of 0.06 g.

**Fig. 19:**
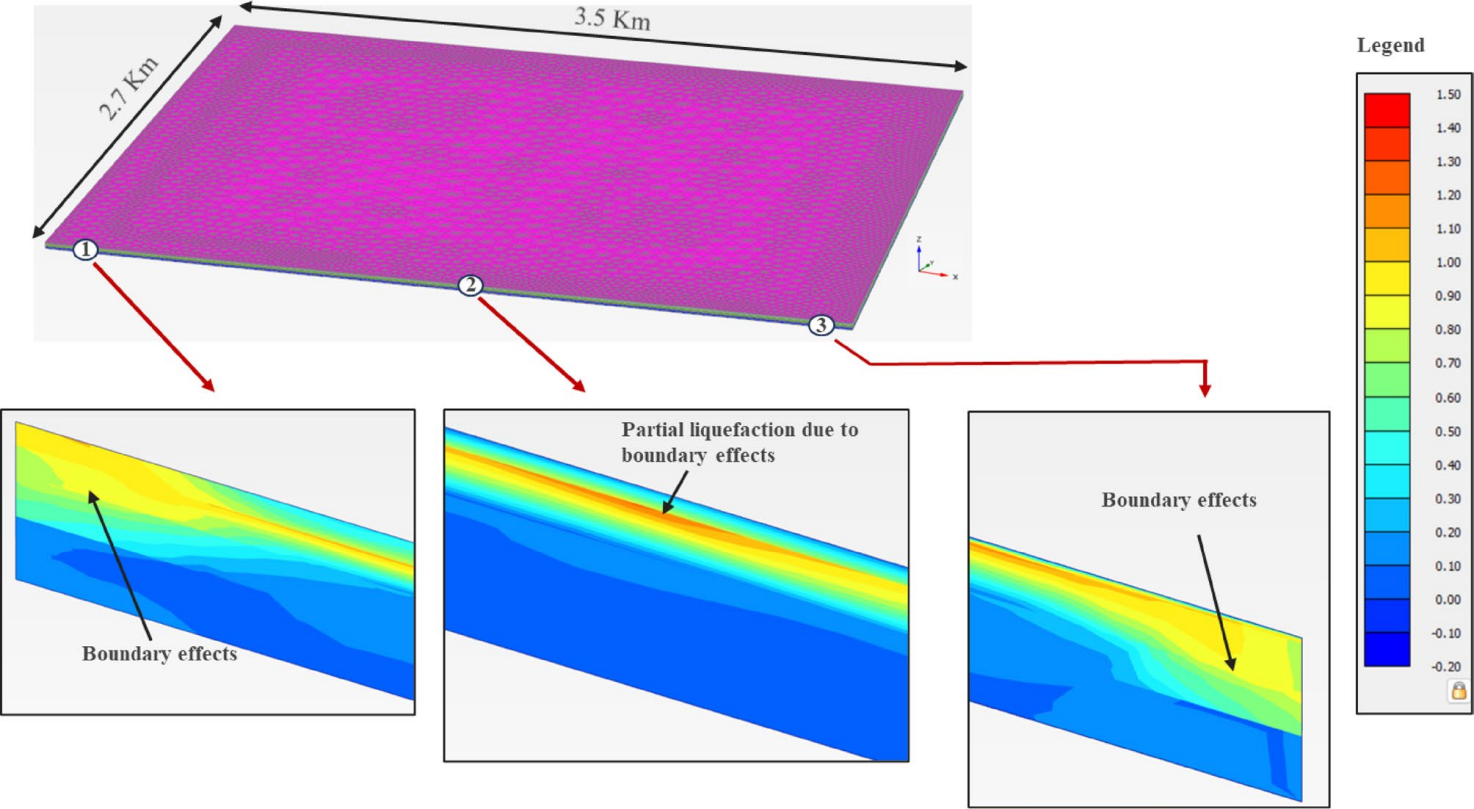
Liquefaction potential of clayey sabkha zone at PGA of 0.10 g.

**Fig. 20:**
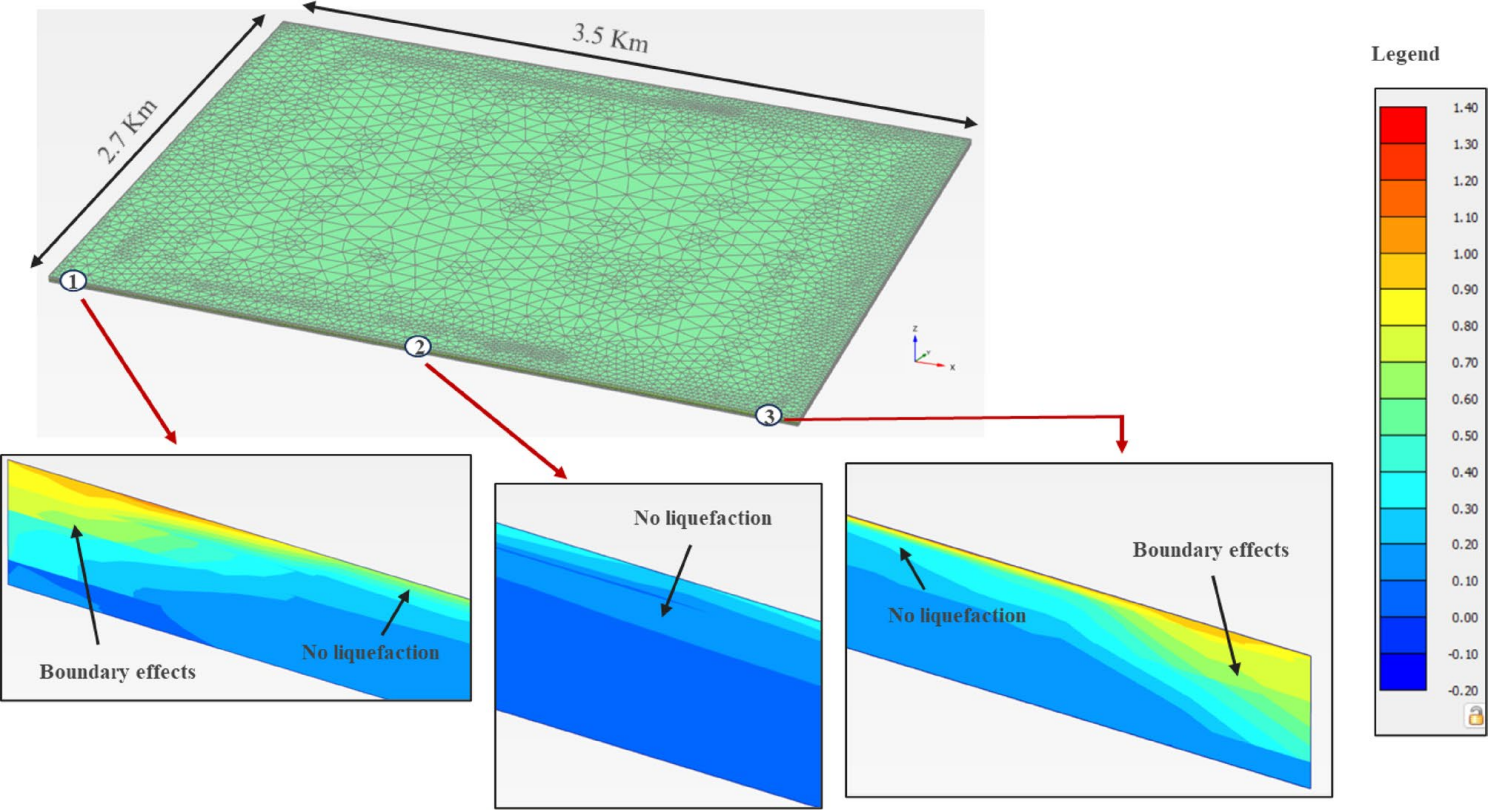
Liquefaction potential of non-sabkha zone at PGA of 0.10 g.

The excess pore-water pressure ratio (r_u_ = u_d_/$\upsigma _{{\mathrm{v}}}^{\prime }$) is usually considered to assess the liquefaction potential; where u_d_ is the dynamic excess pore-water pressure, and $\upsigma _{{\mathrm{v}}}^{\prime }$ is the effective stress. Therefore, the liquefaction potential was assessed in the PLAXIS 3D models using the r_u_ plots, Figs. 18, 19 and 20, for the three zones. Although, all zones with r_u_ > 1.0 are theoretically liquefiable, an experimental value of 0.7, from the literature^71^, has also been imposed, as a safety measure. It is evident from the results that there is no probability of liquefaction in the non-sabkha and clayey sabkha zones at PGA of 0.06 g, and even at higher PGA values of 0.10 g. But sandy sabkha tend to liquefy at a PGA of 0.060 g. It appears from the results that the liquefaction potential increases due to pore pressure restrictions in the salt-saturated pores of sandy the sabkha soils. The salt-saturated pore water pressure cannot be dissipated quickly, due to low permeability, as evident from the laboratory permeability results presented in "Field and laboratory results" section. The salt precipitated as solid particles creates a restriction in the flow paths of the water, which results in low permeability.

Another observation is the liquefaction potential of the non-sabkha zones along their boundaries with the clayey sabkhas (Figs. 18, 19 and 20). In this case, the liquefaction is anticipated along the boundaries where the dissipation of pore pressures is constrained by the interface of the fine-grained/clayey strata. This phenomenon most likely owes to the restriction of pore pressure dissipation along the boundary between sandy and clayey sabkha zones. The fine-grained nature of the clayey sabkhas acts as a low-permeability zone, thereby restricting the dissipation of excess pore pressures developed in the adjacent sandy strata due to seismic activity, which leads to local liquefaction.

**Fig. 21:**
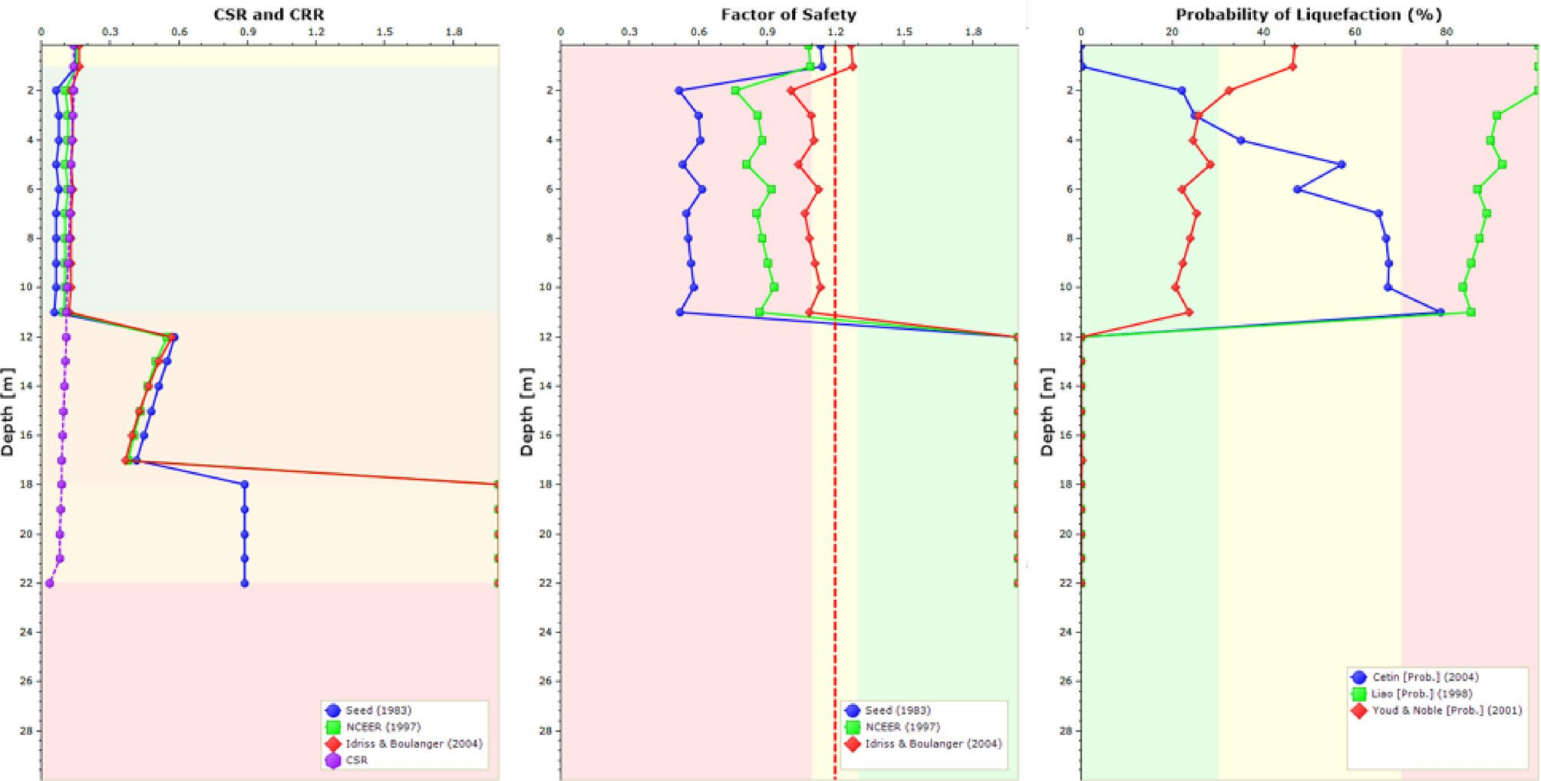
Results of Settle3D for sandy sabkha zone.

For comparison and validation purposes, Fig. 21 presents typical results of cyclic stress ratio, cyclic resistance ratio, probability of liquefaction in different layers, and factor of safety against liquefaction. It indicates that the upper layer, down to 12 m, is liquefiable, as the factor of safety is less than 1. These results are for sandy sabkha zone, and it confirms the results given in Table 12 from PLAXIS 3D.

### Seismic hazard zoning maps

**Fig. 22:**
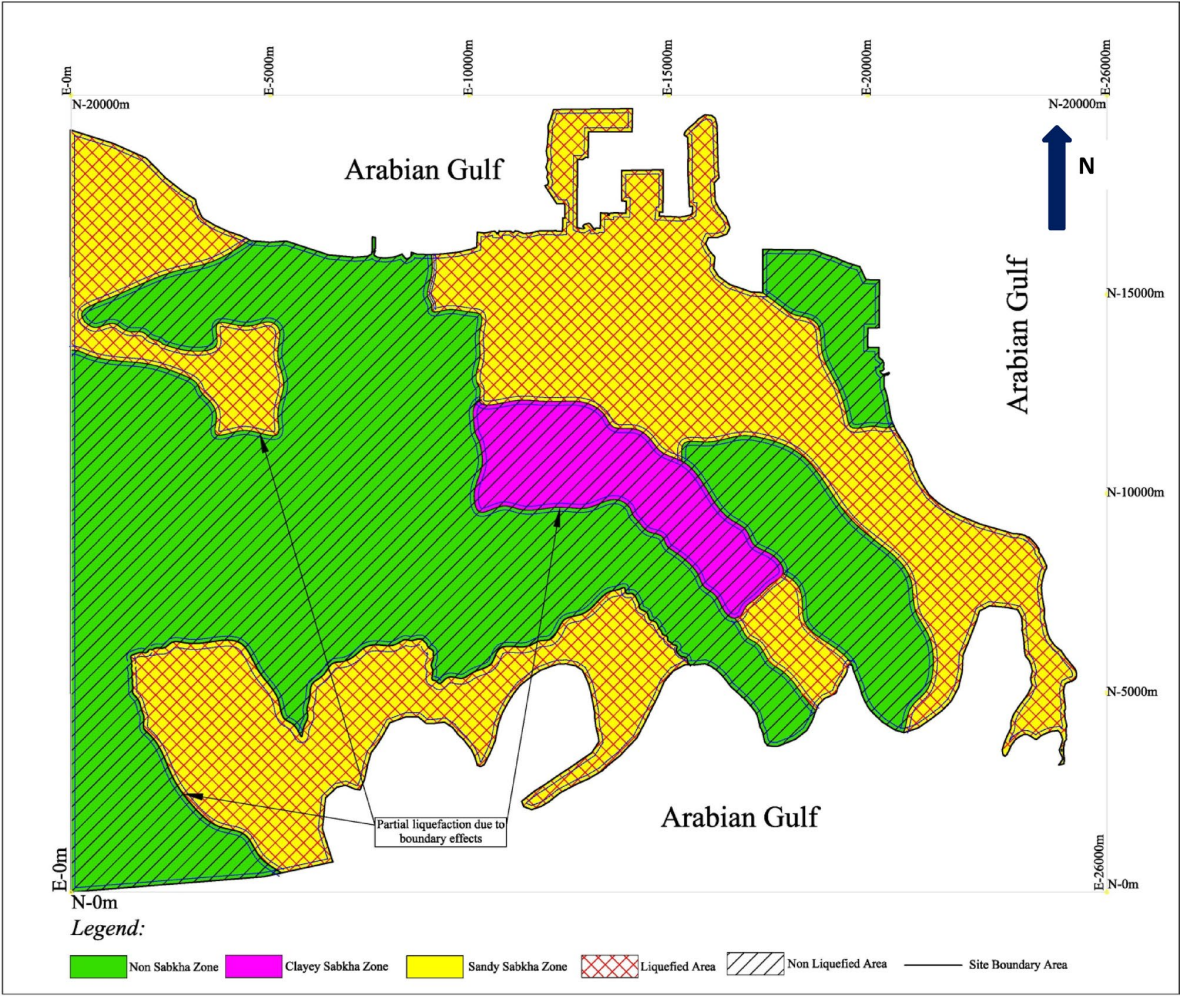
Liquefaction hazard map at PGA of 0.06 g.

**Fig. 23:**
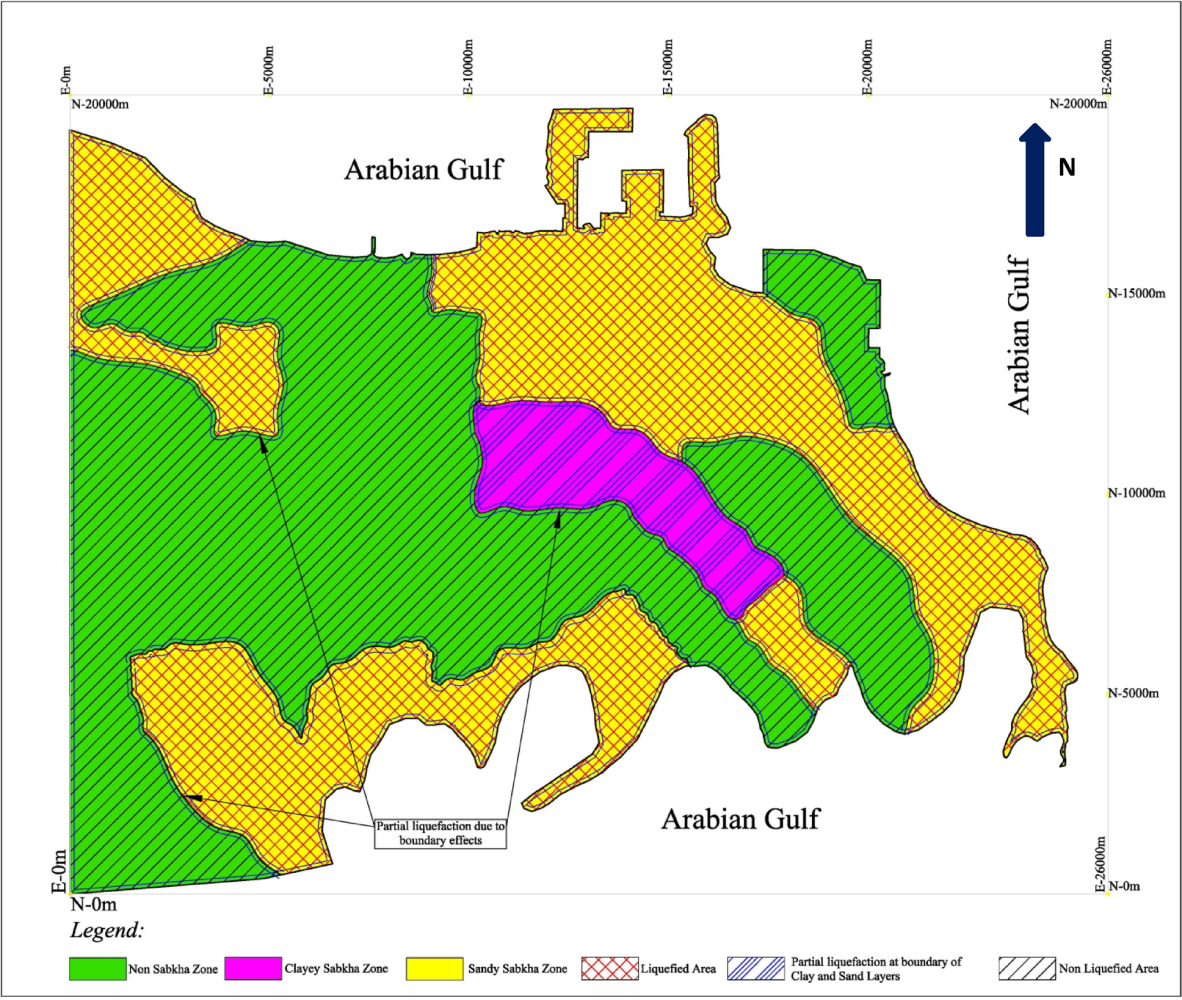
Liquefaction hazard map at PGA of 0.10 g.

As an outcome of this study, the seismic hazard maps, which present the liquefaction potential of various subsurface layers. The liquefaction potential of subsurface strata layers was superimposed on the zoning maps (Fig. 9) for seismic loadings of PGA equals 0.06 and 0.1 g. The results are plotted in Figs. 22 and 23, and the results are summarized in Table 14. In addition to the general liquefaction potential layers, these maps also indicate the liquefiable areas along the borders of the different zones, and along the borders of the different layers in each layer. These narrow strips along the borders would tend to liquefy at a PGA even lower than the one for the rest of the strata. This fact leads to a critical conclusion that all the sandy strata layer confined by fine-grained layer could liquefy during the seismic activity, even if they show no liquefaction potential as a layer by itself. These localized liquefaction potential pockets occur along the boundaries between sandy and clayey layers, where the adjacent low-permeable strata restrict the dissipation of excess pore pressures generated by seismic activity. Therefore, the localized liquefied pockets along the boundaries of various layers should be considered during the design and construction of facilities in these zones.

**Table 14:** The liquefaction potential under different seismic loading conditions.

Zone	Liquefaction potential at PGA of			Localized liquefaction due to boundary effects at PGA of		
	0.06 g	0.08 g	0.10 g	0.06 g	0.08 g	0.10 g
Sandy sabkha	Yes	Yes	Yes	Yes	Yes	Yes
Clayey sabkha	No	No	No	Yes	Yes	Yes
Non-sabkha	No	No	No	Yes	Yes	Yes

## Conclusions and recommendations

This study has led to the following conclusions and recommendations that are limited to the scope of the study.

### Conclusions

1. The site-specific seismic response is not only dependent on the structure and fabric of the soil, but also highly affected by the type of the type of the pore fluid.
2. Anomalous dynamic response of sabkha soils is identified. A more realistic analysis of this anomalous response was further facilitated using 3D modeling and simulations.
3. The sabkha soils are found to have a higher shear modulus compared to equivalent non-sabkha soils. The effect of the salinity of pore water on the dynamic response of soils is established, through an increase in the shear modulus of soils with increasing salt concentrations of the pore water.
4. The 3-D seismic analysis produces more realistic results compared to those obtained from equivalent 1-D models.
5. Liquefaction potential is anticipated in sandy sabkha layers at a design peak ground acceleration (PGA) of 0.06 g. However, no probability of liquefaction has been observed in the clayey sabkha and non-sabkha zones, even at higher PGA values of 0.10 g.
6. There are localized liquefaction potential pockets along the boundaries between sandy and clayey layers. These narrow strips along the borders tend to liquefy at a PGA lower than the one that causes liquefaction for the whole strata.
7. The spectral accelerations (S_s_ and S_l_) have been found to differ from the values specified in local codes.

### Recommendations

1. The discovery of localize liquefiable layers should initiate cautionary measures for the existing infrastructure in these border areas. Moreover, it is recommended to avoid these border areas for any infrastructure in the future.
2. The design of existing structures needs to be verified against the different spectral acceleration values determined in this study; future structures should also be designed against these suggested values.
3. Seismic codes need to be revised using the outcomes from 3-D modelling and simulations, based on specific dynamic parameters of the soil with the actual pore fluids.

## Data Availability

Data generated or analyzed during this study are provided in full within the published article.

## Abbreviations

$\upsigma _{{\mathrm{v}}}^{\prime }$: Effective vertical stress
ASTM: American Society for Testing and Materials
CB: Correction for borehole diameter
CE: Correction for hammer energy efficiency
CN: Overburden correction
CR: Correction for “short” rod length
CS: Correction for non-standardized sampler configuration
D: Damping ratio
FEM: Finite Element Method
g: Acceleration due to gravity
HSsmall: Hardening soil (low strain) constitutive model
K: Coefficient of hydraulic permeability
KFUPM: King Fahd University of Petroleum & Minerals
MASW: Multichannel analysis of surface wave
N: Uncorrected field acquired SPT Blows
N_1_: Overburden corrected SPT blows = N × CN
N_1,60_: Corrected SPT N_1_ = N_1_ × CR × CS × CB × CE
PGA: Peak Ground Acceleration
PSA: Seismic Response Spectral Analysis
PPM: Parts per million
RC: Royal Commission Jubail
RIC: Ras Al Khair Industrial City
r_u_: Excess pore-water pressure ratio
S_1_: 1-Sec spectral acceleration
SAES: Saudi Aramco Engineering Standard
SBC: Saudi Building Code
SGS: Saudi Geological Survey
SPT: Standard Penetration Test
S_s_: Short-period spectral acceleration (0.2 s)
UBSAND: University of British Columbia Sand Constitutive model
u_d_: Dynamic excess pore-water pressure
